# A novel L-RNA aptamer to regulate the pUG fold RNA-induced gene expression *in vivo*

**DOI:** 10.1093/nar/gkaf137

**Published:** 2025-03-08

**Authors:** Shiau Wei Liew, Dong Cao, Riley J Petersen, Samuel E Butcher, Scott G Kennedy, Chun Kit Kwok

**Affiliations:** Department of Chemistry and State Key Laboratory of Marine Pollution, City University of Hong Kong, Kowloon Tong, Hong Kong SAR, 999077, China; Department of Genetics, Blavatnik Institute at Harvard Medical School, Boston, MA 02115, United States; Department of Biochemistry, University of Wisconsin-Madison, Madison, WI 53706, United States; Department of Biochemistry, University of Wisconsin-Madison, Madison, WI 53706, United States; Department of Genetics, Blavatnik Institute at Harvard Medical School, Boston, MA 02115, United States; Department of Chemistry and State Key Laboratory of Marine Pollution, City University of Hong Kong, Kowloon Tong, Hong Kong SAR, 999077, China; Shenzhen Research Institute of City University of Hong Kong, Shenzhen, 518057, China

## Abstract

G-quadruplex (G4) is a guanine-rich secondary structure found in DNA and RNA involved in various biological roles. Recently, a non-canonical RNA G-quadruplex (rG4), known as poly(UG) (pUG) fold, was discovered in *Caenorhabditis elegans*. This unique structure was found to induce RNA interference (RNAi) upon recruitment of RNA-dependent RNA polymerase (RdRP), resulting in trans-generational gene silencing. Herein, we develop a novel L-RNA aptamer, L-apt3.1, that binds to the pUG fold. We uncover that L-apt3.1 consists of a parallel rG4 structural motif, and mutagenesis analysis illustrates that the rG4 motif in L-apt3.1 is essential for pUG fold recognition. We show that L-apt3.1 interacts strongly with pUG fold, and notably, it is the first reported aptamer that can bind to pUG fold *in vitro*. We also demonstrate that L-apt3.1 possesses great biostability in cellular environments and negligible toxicity *in vivo*. Furthermore, we report that L-apt3.1 can interact with pUG *fold in vivo*, and with a comparable performance to the G4 ligand, *N*-methyl mesoporphyrin, in inhibiting gene silencing in *C. elegans*. Overall, we demonstrate the development of pUG fold-targeting L-RNA aptamer for the first time, and show that this new aptamer tool can be applied to control pUG fold-mediated gene expression *in vivo*.

## Graphical Abstract

**Graphical Abstract:**
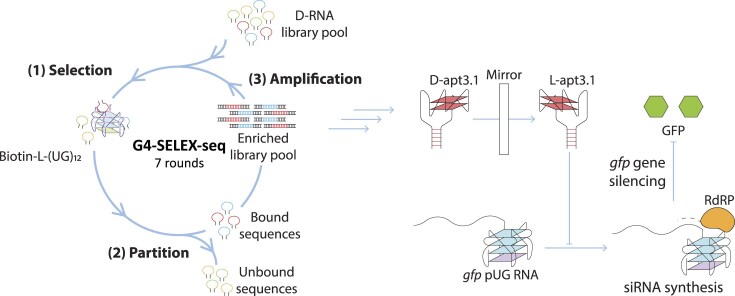

## Introduction

G-quadruplex (G4) structures, formed from guanine (G)-rich DNA or RNA sequences, have emerged as important secondary nucleic acid structures implicated in various biological activities [1–4]. These structures consist of stacked G-quartets, which are planar arrangements of four G bases held together by Hoogsteen hydrogen bonds [2, 3]. G4 structures are typically stabilized by potassium (K^+^) or sodium (Na^+^) ions located between G-quartets [1–3]. G4s have been associated with diseases, including cancers [5, 6], neurological disorders [7, 8], and viral infections [9, 10]; hence, the roles of G4s have been actively explored to further understand the molecular mechanisms by which G4s contribute to disease pathogenesis and to identify potential therapeutic targets [3, 11]. G4 structures exhibit great structural diversity; with a remarkable flexibility and adaptability to adopt different folded configurations, resulting in a wide array of possible structural conformations [12, 13]. On the one hand, canonical G4 structures display well-defined structural motifs, usually characterized with minimally three stacked G-quartets and short loops, with a consensus sequence of G_3+_N_1–7_G_3+_N_1–7_G_3+_N_1–7_G_3+_ [14, 15]. On the other hand, non-canonical G4 structures are composed of more unusual sequence motifs, which possess unique conformational features including long loops [16], bulges [17, 18], and G-vacancies [19, 20].

**Figure 1.**
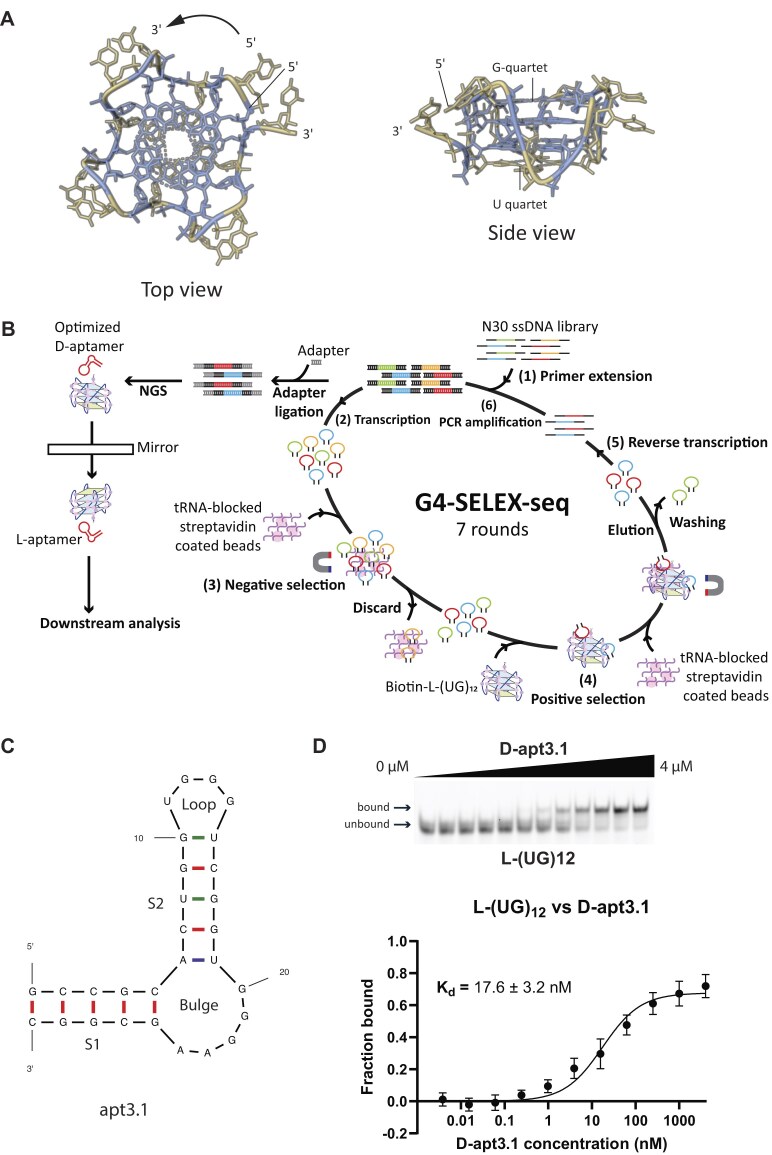
The development of apt3.1 as a pUG fold binding aptamer with nanomolar binding affinity. (**A**) The pUG fold structure (PDB 8TNS) [22]. The G bases are shown in blue, and the U bases are shown in yellow. This unique rG4 motif adopts a left-handed structure composed of three G-quartets and one U quartet. (**B**) The flowchart of G4-SELEX-seq. The ssDNA library pool was converted into its RNA form via (1) primer extension followed by (2) *in vitro* RNA transcription. (3) Negative selection was performed to remove nonspecific sequences, and (4) positive selection was conducted to obtain L-(UG)_12_ binding sequences. The library pool was then (5) reverse transcribed and (6) PCR amplified, and the whole process was repeated for seven rounds. The library pool was then sequenced, and aptamer was selected based on the enrichment and structure for downstream analysis. (**C**) The secondary structure of apt3.1 predicted by Mfold program [37]. The predicted structure consists of two stems of five base pairs each (Supplementary Figs S1 and S2), as well as a 5 nt bulge and a 4 nt loop. (**D**) Binding between FAM-L-(UG)_12_ target and D-apt3.1 aptamer. The Kd value of D-apt3.1 with L-(UG)_12_ was determined to be 17.6 ± 3.2 nM, which is similar to that of D-apt3 (Supplementary Fig. S6A, Kd = 21.9 ± 3.4 nM), indicating that the aptamer trimming and optimization did not affect the target recognition. Data are representative of three independent experiments presented as mean ± standard deviation (SD).

Recently, Roschdi *et al.* discovered a non-canonical G4 structure termed poly(UG) (pUG) fold (Fig. 1A) [21], which consists of repeating units of the uracil-guanine (UG) dinucleotides. The pUG fold structure was determined by X-ray crystallography, and nuclear magnetic resonance spectroscopy and small and wide-angle X-ray scattering (NMR-SAXS-WAXS) in the presence and absence of the G4 ligand, *N*-methyl mesoporphyrin XI (NMM), respectively [21, 22]. NMM interacts with pUG fold by stacking on top of the G-quartet, without perturbing the pUG fold structural conformation [21]. The pUG fold structure consists of structural features distinct from those of other G4 structures. For example, instead of the commonly reported right-handed backbone for the G4 structure, pUG fold has a left-handed backbone, which is formed by the stacking of three G-quartets and one U quartet with bulged Us between the first two G-quartets and U propeller loops connecting the G-tracts (Fig. 1A). Formation of pUG fold requires six K^+^ ions to adopt a compact structure. Studies have reported that a minimum of 11.5 repeats of GU dinucleotides is necessary for the pUG tail to fold into the pUG fold structure [21, 22]. This unique pUG fold structure was first found to be involved with gene regulation in *Caenorhabditis elegans*.

The pUG fold forms from an underlying RNA sequence comprising of perfect UG repeats [23]. The pUG fold was found to induce gene silencing in *C. elegans* via RNA interference (RNAi) [23]. During RNAi, ribonucleotidyltransferase RDE-3 or MUT-2 first adds alternating U and G nucleotides to the 3′ end of target RNAs [23]. This 3′ modification, termed the pUG tail, folds into the pUG fold structure and recruits RNA-dependent RNA polymerase (RdRP), which uses pUG RNAs as templates to synthesize small interfering RNAs (si)RNAs [21, 23]. These siRNAs can further induce pUGylation, which involves the addition of a pUG tail to an mRNA [23]. RNAi-based gene silencing is amplified by repeated cycles of pUG tail and siRNA synthesis, which can result in transgenerational epigenetic inheritance (TEI) [23]. This gene silencing could be inhibited by NMM, which binds to the pUG fold to block the recruitment of RdRP [21]. Despite its notable effects on gene silencing, NMM lacks selectivity towards G4 motifs, where it can recognize parallel dG4s and rG4s [24, 25]. NMM’s moderate affinity for the pUG fold also results in a need for higher NMM concentrations to target the pUG fold [21]. These characteristics impose challenges for the study of the pUG fold structure within biological systems.

Aptamers, also referred to as chemical antibodies, are short single-stranded oligonucleotides folded into unique structure scaffolds which can bind to specific target molecules with high affinity and specificity [26, 27]. Aptamers may offer several advantages over G4 ligands, including specificity, versatility, and stability, which make them potential tools for targeted recognition of diverse G4 motifs. Spiegelmers, a class of modified aptamers, are composed of enantiomers of naturally occurring D-RNA nucleotides, known as L-RNA nucleotides [28–30]. As L-RNA nucleotides exhibit remarkable nuclease resistance, L-RNA aptamers generally possess greater biostability than D-RNA aptamers; therefore, L-RNA aptamers are ideal tools for studying targets of interest *in vitro* and *in vivo* [31–34]. Aptamers are commonly identified via systematic evolution of ligands by exponential enrichment (SELEX), an *in vitro* selection method involving iterative rounds of selection, amplification, and enrichment [35]. Our group previously reported an innovative selection approach for the identification of L-RNA aptamers, G4-systematic evolution of ligands by exponential enrichment-sequencing (G4-SELEX-seq), which combines conventional SELEX with next-generation sequencing (NGS) [36]. In this study, we develop the first pUG fold-binding L-RNA aptamer, L-apt3.1, using G4-SELEX-seq. We demonstrate a strong interaction between L-apt3.1 and the pUG fold structure, with a parallel rG4 motif discovered in L-apt3.1 as the major player in the interaction. Finally, we reported the ability of L-apt3.1 to inhibit gene silencing induced by pUG RNAs *in vivo* in *C. elegans*.

## Material and methods

### Materials

All oligonucleotides, including biotin- and FAM-labelled D-/L-(UG)_12_, D-DNA N30 library template, forward and reverse primers, aptamer candidate templates, as well as other structural constructs, were purchased from IDT, Genewiz or Biosyntech. HiScribe^TM^ T7 High Yield RNA Synthesis Kit (NEB), SuperScript III Reverse Transcriptase (Invitrogen), and KAPA HiFi HotStart ReadyMix (Roche) were used in T7 *in vitro* transcription, reverse transcription and PCR amplification, respectively throughout SELEX. Streptavidin coated magnetic beads (MCE) was used for the pull down of biotin-labelled targets. G4 ligands, including *N*-Methyl-mesophophyrin IX (NMM) (Frontier Scientific or Frontier Specialty Chemicals) and Thioflavin T (ThT) (Solarbio Life Science) were dissolved in DMSO (Acros Organic) prior to use.

### Circular dichroism (CD) assay

A 2 ml of reaction mixture consisting of 5 μM oligo, 10 mM LiCaco buffer (pH 7.0) and 150 mM KCl/LiCl was prepared. The reaction mixture was denatured at 95°C for 5 min and cooled to room temperature. The CD spectroscopy of the reaction mixture was then performed using a Jasco CD J-150 spectrophotometer with a 1 cm path length quartz cuvette. The CD spectra were collected at a 2 nm interval from 220 to 310 nm and were smoothed over 5 nm.

### UV melting spectroscopy

A 2 ml of reaction mixture consisting of 5 μM oligo, 10 mM LiCaco buffer (pH 7.0) and 150 mM KCl/LiCl was prepared. The reaction mixture was denatured at 95°C for 5 min and cooled to room temperature. The UV melting spectroscopy of the reaction mixture was then performed using a Cary 100 UV-vis spectrophotometer or a Cary 3500 UV-Vis Multicell Peltier spectrophotometer with a 1 cm path length quartz cuvette. The UV melting spectra were collected at a 0.5°C interval from 20°C to 95°C and were smoothed over 5°C.

### Thermal difference spectrum

A 2 ml of reaction mixture consisting of 5 μM oligo, 10 mM LiCaco buffer (pH 7.0) and 150 mM KCl/LiCl was prepared. The reaction mixture was denatured at 95°C for 5 min and cooled to room temperature. The absorbance of the reaction mixture was then performed using a Cary 3500 UV-Vis Multicell Peltier spectrophotometer with a 1 cm path length quartz cuvette. The spectra were collected at a 1 nm interval from 220 to 340 nm at 20°C and 90°C, and the thermal difference spectrum (TDS) spectrum was obtained by subtracting the spectra at 20°C with 90°C.

### *In vitro* selection (G4-SELEX-Seq)

Two sets of library pool were designed, one with 30-nucleotide-long randomized sequence (N30 nor) and the other with 30-nucleotide-long randomized sequence with a 30% GC ratio (N30 30% GC). The ratio of A:T:C:G of the low GC library pool was 35:35:15:15. SELEX was conducted with the two sets of library pool in parallel.

The dsDNA library was first generated by preparing a library reaction of 50 μl consisting of 2 μM ssDNA N30 normal/N30 30% GC library template, 3 μM reverse primer, 10 U/μl SuperScript III reverse transcriptase (RT), 1 × Li RT buffer (20 mM Tris-HCl (pH7.5) (Invitrogen), 4 mM MgCl_2_ (Thermo), 1 mM DTT (Invitrogen), 150 mM LiCl (Thermo)) and 1 mM dNTP mixture (Invitrogen). The template was allowed to denature at 75°C and cool down to room temperature before adding reverse transcriptase, and the reaction mixture was incubated at 50°C for 50 min. The dsDNA library was then column purified (Zymoclean Gel DNA Recovery Kit, Zymo Research) and used as the template for T7 *in vitro* RNA transcription according to the protocol from the NEB HiScribe^TM^ T7 High Yield RNA Synthesis Kit. The 40 μl reaction was incubated at 37°C for 3 h and incubated for another 15 min after the addition of 2 U/μl Turbo DNase (Invitrogen). After adding 2 × RNA stopping dye (NEB), the samples were heated at 95°C for 3 min and resolved by 10% denaturing polyacrylamide gel electrophoresis (PAGE) (8 M urea (Thermo) and 1 × Tris borate EDTA (TBE) (Bio-rad)). The bands of desired size were cut and crushed, and were incubated while shaking at 1300 rpm at 4°C overnight in 80 μl/well TEL800 buffer (1 × Tris-EDTA (pH 7.4) (Sigma), 0.8 M LiCl). The gel pieces were removed from the samples (Spin-X Centrifuge Tube Filter, Costar), and the samples were then column purified (RNA Clean and Concentrator-5, Zymo Research) and eluted in nuclease-free water (Invitrogen). The concentration of the purified RNA was measured using aNanoDrop 1000 spectrophotometer (Thermo).

Before the selection step, 750 μg of streptavidin coated magnetic beads (MCE) were washed and incubated with 0.1 mg/ml yeast tRNA (Invitrogen) and selection buffer (25 mM Tris-HCl (pH 7.5), 150 mM KCl (Thermo), 20 mM MgCl_2_) at room temperature for 30 min while shaking at 700 rpm. Another 50 μl of reaction mixture consisting of 1000 pmol of the purified RNA library pool and RNA buffer (50 mM Tris-HCl (pH 7.5), 150 mM KCl) was denatured at 95°C for 5 min and cooled to room temperature. Negative selection was then conducted by adding 250 μg of the tRNA-pre-blocked beads into the library pool mixture and incubated at room temperature for 2 h while shaking at 700 rpm. The streptavidin beads, along with the beads binding non-specific sequences, were then removed.

A target solution consisting of 200 pmol biotin-labelled L-(UG)_12_ and RNA buffer (50 mM Tris-HCl (pH 7.5), 150 mM KCl) was denatured at 95°C for 5 min and cooled to room temperature. The negative selection reaction mixture was then mixed with the target solution, and the mixture was incubated at room temperature for 30 min while shaking at 300 rpm. After the addition of the remaining beads, the mixture was incubated for another 30 min while shaking at 700 rpm. The supernatant consisting of the non-binding sequences was discarded, and the beads were washed with 600 μl of selection buffer for five times before eluting the captured RNA with 200 μl of elution buffer (25 mM NaOH (Sigma), 1 mM EDTA (Fisher)). The eluted RNA was neutralised with 1 M Tris-HCl (pH 7.5) and column purified (RNA Clean & Concentrator-5, Zymo Research).

Subsequently, the purified RNAs were reverse transcribed in a reaction mixture of 60 μl consisting of 0.3 μM reverse primer, 10 U/μl SSIII Reverse Transcriptase, 1 mM each dNTP, 1 × Li RT buffer, which was incubated at 37°C for 15 min. To denature the reverse transcriptase and degrade the remaining RNA template, 3 μl of 2 M NaOH was added to the mixture and incubated at 95°C for 10 min. After that, 15 μl of 1 M Tris-HCl (pH 7.5) was added to neutralise the mixture before column purification (RNA Clean & Concentrator 5, Zymo research). The purified complementary DNA was then PCR amplified (PCR conditions: 95°C for 3 min, 98°C for 20 s, 65°C for 20 s, and 72°C for 20 s, repeat for N cycles depending on the appearance of the enriched band on agarose gel) in a mixture consisting of 0.5 μM forward and reverse primers and 1 × KAPA HiFi HotStart ReadyMix with number of PCR cycles shown in Supplementary Table S2. The amplified dsDNA was then column purified (Zymoclean Gel DNA Recovery Kit, Zymo Research) and used for the next round of selection cycles. The corresponding conditions for each round of selection cycles are listed in Supplementary Table S2.

After the final round of selection cycle, adapter ligation was performed to the amplified dsDNA of rounds 3–7 to attach the adapters used in NGS. The reaction mixture consisting of in 0.5 μM forward and reverse adapter primers and 1 × KAPA HiFi HotStart ReadyMix was PCR amplified for 4 cycles and quantified using Nanodrop spectrophotometer. Around 200 ng of each library pool was mixed together and subjected to NGS by Guangzhou IGE Biotechnology Ltd.

### Sequence analysis and RNA secondary structure prediction

The NGS data was analysed and compared using Microsoft Excel, where the sequences were arranged based on the percentage of reads in each round respectively. The secondary structures of the sequences of the highest frequencies were predicted using Mfold web server [37], and the aptamer candidates were selected based on the enrichment and predicted structure.

G4 prediction analysis consisting of G4Hunter (G4H) and G4 neural network (G4NN) was also performed on the selected aptamer candidates using G4screener v0.3 (http://scottgroup.med.usherbrooke.ca/G4RNA_screener/), with the G4 threshold defined as G4H > 0.9 and G4NN > 0.5 according to the default settings.

### Electrophoretic mobility shift assay

#### Binding assay

A reaction mixture consisting of of 10 nM 5′ FAM labelled D-/L-(UG)_12_, 1 × binding buffer (1 mM MgCl_2_, 25 mM Tris-HCl (pH 7.5), 150 mM KCl) were prepared and denatured at 95°C for 5 min. The reaction mixture was allowed to slow cool to 4°C with a ramp rate of 0.1°C/s. Varying concentrations of L-/D-aptamer were prepared in the same 1 × binding buffer via serial dilution by a factor of 4. The reaction mixture was denatured at 95°C for 5 min and cooled to room temperature for 15 min. The two reaction mixtures were then added together to a final volume of 10 μL and incubating at 37°C for 30 min. After addition of glycerol solution to a final concentration of 10%, the samples were loaded onto an 8% (19:1, acrylamide/bis-acrylamide) native polyacrylamide gel consisting of 50 mM potassium acetate (Amethyst Chemicals), 1 mM MgCl_2_, and 25 mM Tris-HCl (pH 7.5) and electrophoresed at around 150 V at 4°C for 30 min. The gel was scanned with the Cy2 filter of an Amersham Typhoon RGB laser-scanner (Cytiva) and quantified by ImageJ software. The curve fitting and Kd value determination was performed with GraphPad Prism using one-site specific binding model.

#### Binding selectivity

A 10 μl of reaction mixture consisting of 10 nM or 20 nM 5′ FAM labelled target and 1 × binding buffer (1 mM MgCl_2_, 25 mM Tris-HCl (pH 7.5), 150 mM KCl) was prepared, and 100 nM L-aptamer was added in the samples. Another set of reaction mixture was prepared without the addition of apt3.1. Both reaction mixtures were denatured at 95°C for 5 min and incubated at 37°C for 30 min. After addition of glycerol solution to a final concentration of 10%, the samples were loaded onto an 8% (19:1, acrylamide/bis-acrylamide) native polyacrylamide gel consisting of 50 mM potassium acetate, 1 mM MgCl_2_, and 25 mM Tris-HCl (pH 7.5) and electrophoresed at around 150 V at 4°C for 30 min. The gel was scanned with the Cy2 filter of an Amersham Typhoon RGB laser-scanner (Cytiva) and quantified by ImageJ software.

#### Competition assay for D-(UG)_12_ against L-apt3.1 and NMM

A reaction mixture consisting of of 10 nM 5′ FAM labelled D-(UG)_12_ and 1 × binding buffer (1 mM MgCl_2_, 25 mM Tris-HCl (pH 7.5), 150 mM KCl) was prepared. The reaction mixture was denatured at 95°C for 5 min and slow cooled at a ramp rate of 0.1°C/s. Another reaction mixture consisting of 250 nM L-apt3.1 was prepared in the same 1 × binding buffer and denatured at 95°C for 5 min before cooling to room temperature for 15 min. Both reaction mixtures were then added together with varying concentrations of NMM prepared via serial dilution by a factor of 5 to a final volume of 10 μl, and the reaction mixture was incubated at 37°C for 30 min. After addition of glycerol solution to a final concentration of 10%, the samples were loaded onto an 8% (19:1, acrylamide/bis-acrylamide) native polyacrylamide gel consisting of 50 mM potassium acetate, 1 mM MgCl_2_, and 25 mM Tris-HCl (pH 7.5) and electrophoresed at around 150 V at 4°C for 30 min. The gel was scanned with the Cy2 filter of an Amersham Typhoon RGB laser-scanner (Cytiva) and quantified by ImageJ software. The curve fitting and IC50 value determination was performed with GraphPad Prism using log(inhibitor) versus response (three parameters).

#### Displacement assay for D-(UG)_12_ against NMM and L-apt3.1

A reaction mixture consisting of 10 nM 5′ FAM labelled D-(UG)_12_ and 1 × binding buffer (1 mM MgCl_2_, 25 mM Tris-HCl (pH 7.5), 150 mM KCl) was prepared. The reaction mixture was denatured at 95°C for 5 min and slow cooled at a ramp rate of 0.1°C/s. The reaction mixture was then added with 500 nM NMM, and the D-(UG)_12_-NMM mixture was incubated at 37°C for 30 min. Varying concentrations of L-apt3.1 were prepared via serial dilution by a factor of 5, denatured at 95°C for 5 min and cooled to room temperature for 15 min. After 30 min incubation of the D-(UG)_12_-NMM mixture, the mixture was added into the L-apt3.1 to a final volume of 10 μl. The reaction mixture was further incubated at 37°C for 30 min. After addition of glycerol solution to a final concentration of 10%, the samples were loaded onto an 8% (19:1, acrylamide/bis-acrylamide) native polyacrylamide gel consisting of 50 mM potassium acetate, 1 mM MgCl_2_, and 25 mM Tris-HCl (pH 7.5) and electrophoresed at around 150 V at 4°C for 40 min. The gel was scanned with the Cy2 filter of an Amersham Typhoon RGB laser-scanner (Cytiva) and quantified by ImageJ software.

### Microscale thermophoresis

A reaction mixture consisting of of 20 nM 5′ FAM labelled D-/L-(UG)_12_, 1 × binding buffer (1 mM MgCl_2_, 25 mM Tris-HCl (pH 7.5), 150 mM KCl) were prepared and denatured at 95°C for 5 min. The reaction mixture was allowed to slow cool to 4°C with a ramp rate of 0.1°C/s. Varying concentrations of L-/D-aptamer were prepared in the same 1 × binding buffer via serial dilution by a factor of 2. The reaction mixture was denatured at 95°C for 5 min and cooled to room temperature for 15 min. The two reaction mixtures were then added together to a final volume of 10 μL and incubated at 37°C for 30 min. Microscale thermophoresis (MST) binding assay was then performed using a Monolith NT. 155 (NanoTemper) with ‘nano-blue’ channel with capillary tubes. The curve fitting and Kd value determination was performed using MO.Affinity Analysis software.

### G4 ligand enhanced fluorescence spectroscopy

A 100 μl of reaction mixture consisting of 1 μM aptamer, 1 μM G4 ligand (NMM or ThT), 10 mM LiCaco buffer (pH 7.0) and 150 mM KCl/LiCl was prepared. The reaction mixture was denatured at 95°C for 5 min and cooled to room temperature before the addition of the G4 ligand. The fluorescence spectroscopy of the reaction mixture was then performed using a HORIBA FluoroMax-4 fluorescence spectrophotometer with a 1 cm path length quartz cuvette. For ThT ligand, the emission spectra were collected from 440 to 700 nm, and the excitation wavelength was set to 425 nm. For NMM ligand, the emission spectra were collected from 550 to 750 nm, and the excitation wavelength was set to 394 nm. The entrance and exit slits were set to be 5 and 2 nm, respectively, with the data collected at a 2 nm interval.

### 1D NMR

For NMR analysis, L-apt3.1 was 920 μM in 100 mM KCl and 20 mM potassium phosphate buffer pH 7.0. The sample was heated to 95°C for 5 min and cooled to room temperature. 10% D2O and 20 μM 4,4-dimethyl-4-silapentane-1-sulfonate (DSS) were added and the final sample volume was 300 μl. NMR data were collected on a Bruker Avance III HD 600 MHz spectrometer equipped with a TCI cryogenic probe. All spectra were recorded at 293 K. The 1D spectrum used a gradient echo with a selective proton pulse centred on the imino protons. Chemical shifts were referenced to the DSS internal standard set to 0 ppm. The DSS signal was observed in a reference 1D NMR spectrum that used a non-selective proton pulse and water suppression using excitation sculpting with gradients. NMR data were processed using Mnova software.

### NMM staining gel

A 10 μl of reaction mixture consisting of 2.5 μM (UG)_12_ mutants, 150 mM KCl, and 10 mM Tris-HCl (pH 7.5) was prepared. The reaction mixture was denatured at 95°C for 5 min and allowed to cool to 4°C. After addition of glycerol solution to a final concentration of 10%, the samples were loaded onto a 10% (19:1, acrylamide/bis-acrylamide) native polyacrylamide gel consisting of 50 mM potassium acetate and 25 mM Tris-HCl (pH 7.5), and electrophoresed at 100 V at 4°C for 1 h. The gel was then stained with 1 μg/mL NMM for 30 min and visualized with the Alexa 488 filter of a Bio-Rad ChemiDoc Touch imaging system, followed by staining with 1 × SYBR gold for 5 min and scanning with the SYBR gold filter of a Bio-Rad ChemiDoc Touch imaging system.

### NMM binding assay

Varying concentrations of D-(UG)_12_ were prepared via serial dilution by a factor of 2 in 1 × binding buffer (1 mM MgCl_2_, 25 mM Tris-HCl (pH 7.5), 150 mM KCl) and denatured at 95°C for 5 min and slow cooled at a ramp rate of 0.1°C/s. After the addition of 100 nM NMM to a final volume of 50 μl, the reaction mixture was then incubated at 37°C for 30 min. the fluorescence spectroscopy of the reaction mixture was performed with a Molecular Devices SpectraMax ID5 Microplate Reader and a 384-well black plate. The excitation wavelength was set to 394 nm, and the fluorescence signals were analysed at 610 nm. The curve fitting and Kd value determination was performed with GraphPad Prism using one-site specific binding model.

### Stability test

A 10 μl of reaction mixture consisting of 100 nM D-/L-apt3.1 and 5% fetal bovine serum (FBS) (Gibco) was prepared. The reaction mixture was incubated at room temperature for 0 to 120 min before adding 2 × RNA stopping dye (NEB). The mixture was then heated at 95°C for 3 min and resolved by 10% denaturing PAGE (8 M urea and 1 × TBE). The gel was stained with 1 × SYBR gold for 5 min and visualized with the SYBR gold filter of a Bio-Rad ChemiDoc Touch imaging system.

### pUG RNA injection for the study of gene silencing activity

pUG RNA injection was performed as previously [21]. NMM’s concentration was determined by absorbance at 379 nm using extinction coefficient 1.45 × 105 M^−1^·cm^−1^ [38]. *In vitro* transcribed gfp pUG RNA (final concentration 0.8 μM) containing 369 nt GFP coding sequences appended by (UG)_18_ was mixed with L-apt3.1, D-apt3.1 or NMM (final concentration 50 μM/5 μM) in annealing buffer (20 mM Tris pH 7.0, 100 mM KCl). The mix was then heated to 90°C for 3 min and slowly cooled to 25°C at −0.1°C/sec. For (CA)_18_ DNA oligo, it was added to pUG RNA before annealing or after annealing. Then injection marker plasmid PCFJ90 (Addgene, #19 327, *myo-2p::mCherry*) was added to a final concentration of 2.5 ng/μl. This plasmid was used to label F1 worms that have incorporated the injection mix because worms expressing this plasmid show mCherry in the pharynx. The pUG RNA mix was injected into the gonads of young adult worms (YY1581 [*rde-1(ne219); mjIs31(pie-1p::gfp::h2b)*]), which express GFP in the nuclei of mature oocytes. Injected individual P0 worms were transferred onto separate nematode growth media (NGM) plates seeded with OP50 bacteria. After five-day culture at 20°C, young adult F1 worms were washed off with M9 buffer (3 g/L KH_2_PO_4_, 6 g/L Na_2_HPO_4_, 5 g/L NaCl, 1 mM MgSO_4_) containing 0.1% Tween-20 (Sigma) and 1mM (-)-Tetramisole hydrochloride (Sigma), which prevents worm from moving. Anesthetized worms were mounted onto slides and the silencing of GFP in the germline was scored using an inverted fluorescent microscope (Zeiss, Axio Observer.Z1) with either a 10 × or 20 × objective lens. Only injection marker positive F1s were counted. Injected P0 worms that gave less than 10 injection marker positive F1s were excluded from analysis.

For brood size assay, P0 worms were removed from NGM plates 45 h after injection. Then the numbers of F1 progeny were counted 3 days later. Damaged/dead P0 worms were excluded.

## Results

### Identification of D-RNA aptamer that targets L-(UG)_12_

To identify an L-RNA aptamer specifically targeting pUG fold, we employed pUG fold sequence (D-(UG)_12_) to comprise 12 repeating units of UG dinucleotides. To ensure the reaction conditions used for experiment facilitates the proper folding of pUG folds, we first performed CD, UV melting and TDS assays on the D-(UG)_12_. The CD spectrum showed a negative peak at around 245 nm, a positive doublet at 265 nm and 285 nm, and a negative peak at 304 nm (Supplementary Fig. S1A), which are signatures of a pUG fold structure [21]. While the negative peak at 245 nm indicates a parallel topology, the remaining peaks suggest that the pUG fold structure is indeed structurally distinct from other known G4s. Besides, the UV melting spectrum at a wavelength of 295 nm illustrated a hypochromic shift, with the melting temperature (Tm) determined to be 50.5°C (Supplementary Fig. S1B). Additionally, we also performed the TDS spectrum, which compare the absorbance of the folded and unfolded states of nucleic acid structures [39]. The results showed positive peaks at 247 and 274 nm, as well as a negative peak at 297 nm, which are signatures of G4 folding (Supplementary Fig. S1C). These results demonstrated the unique spectral characteristics of pUG fold, confirming that the sequence and the reaction condition do not affect the folding of pUG fold structure. The D-(UG)_12_ was then converted into its L-RNA form, and a biotin molecule was attached to its 5′ end for streptavidin coated magnetic bead pulldown during SELEX. We then further assessed the formation of pUG fold structure of L-(UG)_12_ using the same approaches, and the results suggested an identical topology in the enantiomeric form (Supplementary Fig. S2). We performed G4-SELEX-seq using the designed biotin-L-(UG)_12_ and D-DNA library templates (Fig. 1B) (Supplementary Table S1). It is crucial for the library to be in its D-form during SELEX as the polymerases employed in different steps of SELEX could not recognize L-DNA/L-RNA. To obtain L-(UG)_12_ binding aptamer candidates, seven rounds of SELEX were performed on two library pools, N30 nor library (A:T:C:G = 25:25:25:25) and N30 30% GC library (A:T:C:G = 15:15:35:35). The selection conditions were progressively intensified to retain the stronger binders (Supplementary Table S2). To eliminate non-specific sequences, negative selection was performed in the presence of the beads, and the beads-bound sequences were removed followed by positive selection with the biotin-L-(UG)_12_ under defined conditions (Supplementary Table S2). Unbound sequences were then removed, and the retained sequences were amplified for subsequent rounds of selection. After seven rounds of selection cycles, the enriched D-RNA pool was subjected to NGS (Fig. 1B).

The NGS data revealed enrichment for some sequences throughout the selection cycles. We analysed the sequences based on their enrichment profiles and Mfold predicted secondary structure [37]. The secondary structures with and without the fixed linker regions were compared, with preferences for sequences adopting a similar structure regardless the presence or absence of linker. Based on the analyses, we shortlisted nine aptamer candidates (D-apt1-9) from both library pools for further evaluation (Supplementary Table S3). D-apt1 was selected as it is the most enriched sequence in the final round of N30 nor library, while other aptamer candidates (D-apt2-9) were selected based on their enrichment and structure (Supplementary Fig. S3).

To assess the binding between L-(UG)_12_ and the aptamer candidates, we first designed a FAM-labelled L-(UG)_12_ (FAM-L-(UG)_12_) and verified the folding of pUG fold structure in FAM-L-(UG)_12_ using CD and UV melting assays. Both CD and UV melting assays showed a similar result with the unlabelled L-(UG)_12_ (Supplementary Fig. S4), indicating the presence of the pUG fold structure. We then performed an initial binding test on the aptamer candidates towards FAM-L-(UG)_12_ using electrophoretic mobility shift assay (EMSA). All aptamer candidates showed binding to L-(UG)_12_ to different extents (Supplementary Fig. S5), with D-apt3, 4, 6, and 7 showing a stronger binding among all candidates. The full binding between L-(UG)_12_ and these candidates were then performed using EMSA, with the Kd value of 21.9 ± 3.4 nM, 51.3 ± 9.5 nM, 105.3 ± 23.6 nM, and 39.9 ± 5.3 nM for D-apt3, 4, 6, and 7, respectively (Supplementary Fig. S6). Due to the set up and the sensitivity of the assay, we wish to note that the reported Kd should be considered as apparent Kd values instead [40]. Based on the results, we selected D-apt3 for further study as it was found to have the strongest binding with L-(UG)_12_. Using Mfold program, the secondary structure of D-apt3 was predicted to be a stem-loop structure with a bulge of five nucleotides within the stem and one nucleotide flanking at the 3′ end (Supplementary Fig. S3). To minimize the length and to optimize D-apt3 for downstream experiments, we first removed the flanking nucleotide at the 3′ end as it is likely unnecessary for the interaction with pUG fold. Besides, to reduce the potential formation of alternative secondary structures by the four consecutive guanine residues in the stem region 1 (S1), we further inverted the middle two GC bases in the stem region to form a construct we referred to as apt3.1 (Fig. 1C). The binding ability of D-apt3.1 is comparable to that of D-apt3, with a Kd value 17.6 ± 3.2 nM using EMSA (Fig. 1D), suggesting that the structure of S1, rather than the sequence, is important for the L-(UG)_12_ binding. We also performed MST to verify the binding, and the Kd value was calculated to be 33.5 ± 3.1 nM and 34.3 ± 4.8 nM for D-apt3 and D-apt3.1 respectively (Supplementary Fig. S7). As D-apt3.1 has a shorter length (29 nt) and a reduced possibility to form alternative secondary structures, we selected D-apt3.1 for further analysis on its structure and ability to target L-(UG)_12_. Overall, we identified D-apt3 as the strongest binding aptamer candidate toward L-(UG)_12_, and obtained D-apt3.1 as the final construct after further trimming and optimization of the aptamer.

### Biophysical analysis reveals the presence of rG4 structure in D-apt3.1

We further analysed the sequence of D-apt3.1, and found four sets of consecutive guanine bases (G_9_-G_10_, G_12_-G_14_, G_17_-G_18_, G_20_-G_22_), suggesting the potential formation of rG4 structure within D-apt3.1. To verify the presence of rG4 structure, we first conducted a computational G4 prediction using G4RNA screener web interface [41], which incorporates algorithms including G4Hunter and G4NN. G4Hunter evaluates G4 propensity based on the G-richness and G-skewness of a sequence [42], while G4NN assesses the similarity of a sequence to known G4 sequences [41]. Interestingly, G4H and G4NN provided divergent prediction outcomes for the rG4 formation in D-apt3.1. From the prediction results, G4H suggested the absence of an rG4 structure, with a score of 0.8276, which is below the default threshold (>0.9). G4NN predicted the presence of an rG4 structure in D-apt3.1, with a score of 0.7407, which is above the default threshold (>0.5).

**Figure 2.**
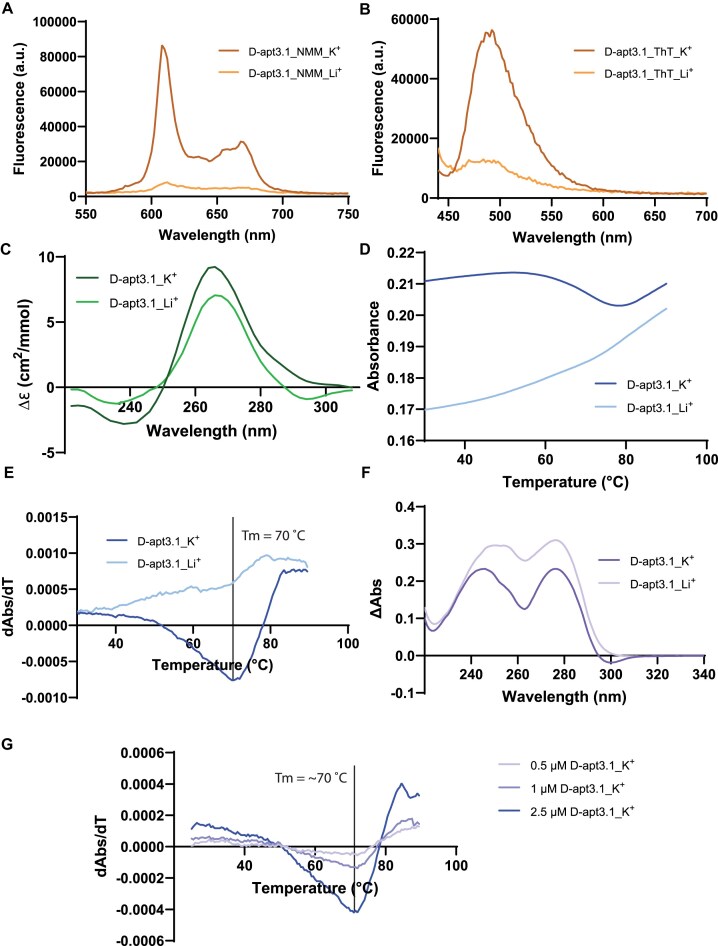
Spectroscopic analysis uncovers the formation of an rG4 structure in D-apt3.1 aptamer. (**A**) NMM- and (**B**) ThT-enhanced fluorescence spectra of D-apt3.1 under 150 mM K^+^ and Li^+^ conditions. The fluorescent intensities of NMM and ThT were found to be higher for apt3.1 under K^+^ condition than Li^+^ condition, which supports the presence of rG4 structure in apt3.1. (**C**) CD spectrum of D-apt3.1 under 150 mM K^+^ and Li^+^ conditions. The difference in CD spectra between K^+^ and Li^+^ condition, as well as the characteristic CD profile in K^+^ condition, with a negative peak at 244 nm and a positive peak at 266 nm, suggest a parallel rG4 structure in D-apt3.1. (**D**) The UV melting curve of D-apt3.1 under 150 mM K^+^ and 150 mM Li^+^ conditions at 295 nm. The decrease of the absorbance under K^+^ condition indicates the denaturation of the rG4 structure of D-apt3.1. (**E**) Normalized first derivative of the UV melting of D-apt3.1 under 150 mM K^+^ and 150 mM Li^+^ conditions. The hypochromic shift monitored at the absorbance wavelength 295 nm for K^+^ condition, but not in Li^+^ condition, indicating the formation of a thermostable rG4 motif under K^+^ condition. From the analysis, the melting temperature (Tm) was found to be 72°C under 150 mM K^+^ condition. (**F**) TDS spectrum of D-apt3.1 under 150 mM K^+^ and 150 mM Li^+^ conditions. The positive peaks at 246 and 276 nm, and the negative peak at 300 nm under K^+^ condition supports the presence of rG4 structure in D-apt3.1. (**G**) Concentration dependent UV melting spectra of D-apt3.1 under K^+^ conditions. The Tm was found to be around 70°C for all concentrations, suggesting an intramolecular structure in D-apt3.1.

To experimentally validate if rG4 structure is present in D-apt3.1, we performed multiple spectroscopic assays under both K^+^ and Li^+^ conditions. We first conducted G4 ligand enhanced spectroscopy using two well-known G4 ligands, NMM and Thioflavin T (ThT), which typically yields higher fluorescence signals in K^+^ conditions as compared to Li^+^ conditions when a G4 structure is present. Our results showed a higher fluorescence signal in the K^+^ condition, suggesting the presence of an rG4 structure in D-apt3.1 (Fig. 2A and B). Since G4 ligands commonly stabilize G4, and have the potential to induce G4 to fold, we further performed label-free experiments to verify the presence of rG4 structure. Firstly, we performed CD spectroscopy to assess the rG4 formation (Fig. 2C). We observed a higher signal under K^+^ condition, further confirming the presence of an rG4 structure in D-apt3.1. A negative peak at 244 nm and a positive peak at 266 nm were observed, suggesting a parallel topology of the rG4 structure. The much lower CD signal and the shift of the negative peak to 230 nm were due to the abolishment of rG4 structure conformation in D-apt3.1 under Li^+^ condition. Additionally, we conducted UV melting assay at a wavelength of 295 nm in K^+^ and Li^+^ conditions to further assess the rG4 formation. We observed a decrease of absorbance under K^+^ condition, which is due to the denaturation of the rG4 structure (Fig. 2D). The normalized first derivative of the UV melting curve demonstrates a hypochromic shift under K^+^ condition, indicating the presence of an rG4 structure in D-apt3.1 under K^+^ condition (Fig. 2E). The melting temperature (Tm) of D-apt3.1 was determined to be 70°C, providing strong evidence for the thermostability of the rG4 structure at physiological-relevant temperature (Fig. 2E). This shift was not found under Li^+^ condition, which is due to the absence of rG4 formation in D-apt3.1 under that condition. Finally, we performed TDS assay by comparing the UV absorbance spectra of D-apt3.1 at 20°C and 95°C to examine the presence of rG4 structure. The spectrum reveals positive peaks at 246 and 276 nm as well as a negative peak at 300 nm under K^+^ condition, which are signatures of a G4 structure, further supporting the rG4 structure formation in D-apt3.1 (Fig. 2F).

Given that G4 structures potentially form high-order structures, we are intrigued to study the molecularity of D-apt3.1. We assessed the structure of D-apt3.1 using UV melting assays with different D-apt3.1 concentrations (0.5, 1, 2.5 μM). The Tm of D-apt3.1 at these concentrations were determined to be around 70°C (Fig. 2G), which is the same as assessed in Fig. 2D (5 μM D-apt3.1). The consistency in the Tm value suggests that D-apt3.1 can fold into an intramolecular rG4 under physiological potassium ion conditions. In short, we reported that D-apt3.1 consists of a G-rich sequence, and further analyses confirmed the presence of a thermostable parallel rG4 structure in D-apt3.1 under physiological-relevant potassium ion and temperature conditions.

### Mutagenesis analysis shows the rG4 structure in D-apt3.1 is essential for recognizing (UG)_12_

**Figure 3.**
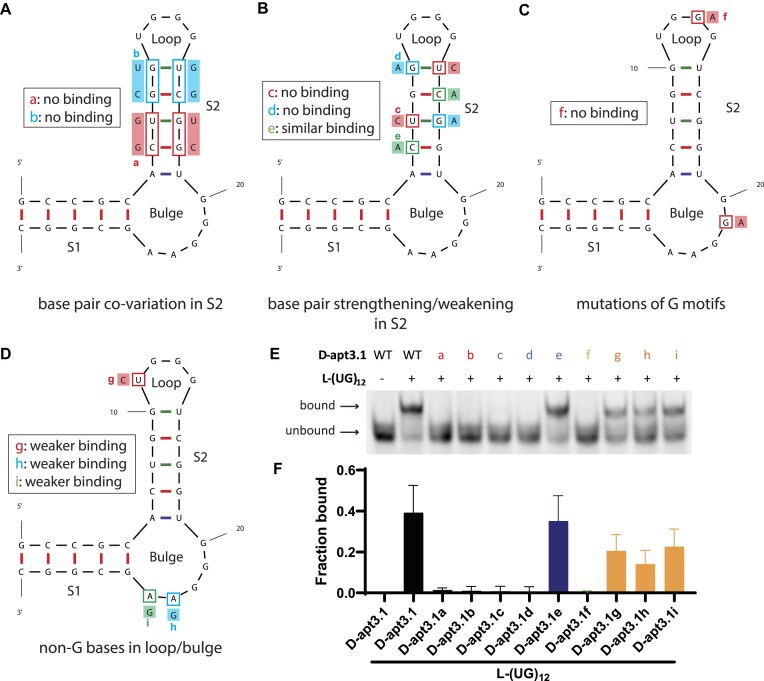
Mutagenesis analysis demonstrates the significance of rG4 motif in apt3.1 for (UG)_12_ recognition. (**A**) Base-pair co-variation in S2, with the base pairs inverted in S2 stem region. The first two base pairs (C_7_-G_18_, U_8_-G_17_) were inverted for D-apt3.1a and the second two base pairs (G_9_-C_16_, G_10_-U_15_) were inverted for D-apt3.1b. The inversion of base pairs preserved the S2 stem structure with a different sequence, and from the binding assay the interaction was abolished for both constructs, suggesting a sequence-specific effect in S2 region. (**B**) Base-pair strengthening or weakening in S2, with the base pairs strengthened (GU to GC/AU) or weakened (GC to GA). D-apt3.1c and D-apt3.1d consist of the strengthened base pairs (D-apt3.1c: U_8_-G_17_ to C_8_-G_17_, G_10_-U_15_ to G_10_-C_15_; D-apt3.1d: U_8_-G_17_ to U_8_-A_17_, G_10_-U_15_ to A_10_-U_15_) in S2 region, which resulted in no binding. This is caused by the disruption of rG4 structure conformation in D-apt3.1. D-apt3.1e consists of weakened base pair (C_7_-G_18_ to A_7_-G_18_, G_9_-C_16_ to G_9_-A_16_), and the binding was found to be similar as WT D-apt3.1, further supporting the need of rG4 structure motif for (UG)_12_ recognition. (**C**) G-to-A substitution of the middle Gs in the GGG runs (G_21_ to A_21_), which leads to the disruption of the rG4 structure (D-apt3.1f), hence resulting in no binding towards L-(UG)12. (**D**) Single nucleotide mutations of the non-Gs in the loop and bulge. D-apt3.1g contains a U-to-C substitution in the loop (U_11_ to C_11_), while D-apt3.1h and D-apt3.1i contain A-to-G substitutions in the bulge (D-apt3.1h: A_23_ to G_23_; D-apt3.1i: A_24_ to G_24_). The binding was weakened upon mutation, indicating that the nucleotides might induce conformational changes of D-apt3.1. (**E** and **F**) Mutagenesis analysis with EMSA, where the (UG)_12_ binding of the mutants was compared to the wild-type D-apt3.1. The mutants that likely reduce the rG4 structure conformation in apt3.1 showed no binding to (UG)_12_, while the mutants that do not directly affect the rG4 structure showed less effect towards the binding. Among the four mutants that bind to L-(UG)_12_, including D-apt3.1e, D-apt3.1g, D-apt3.1h, and D-apt3.1i, D-apt3.1e exhibits a similar binding as D-apt3.1, while the remaining have a weaker binding.

**Figure 4.**
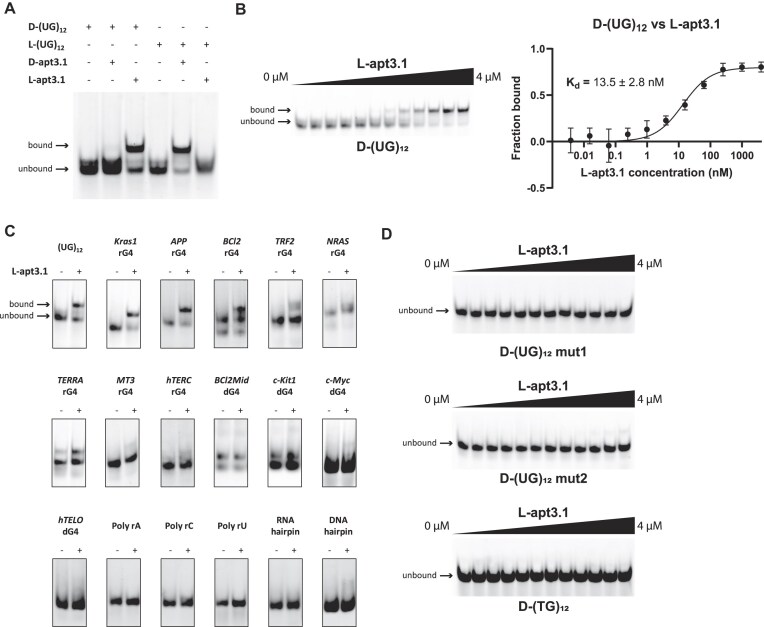
Binding assays reveal that L-apt3.1 preferentially interacts with the intended D-(UG)_12_ target and a few other rG4 motifs. (**A**) Enantiomeric test of apt3.1. D-apt3.1 only binds L-(UG)_12_ and vice versa, indicating the (UG)_12_–apt3.1 interaction is enantiomeric-specific. (**B**) Binding of FAM-D-(UG)_12_ vs L-apt3.1 via EMSA. The Kd value is determined to be 13.5 ± 2.8 nM, which is similar to that of L-(UG)_12_ and D-apt3.1 (Fig. 1D, Kd = 17.6 ± 3.2 nM). Data is representative of three independent experiments presented as mean ± s.d. (**C**) Binding selectivity of L-apt3.1 against FAM labelled on- and off-target. L-apt3.1 does not bind to any dG4 and non-G4 motifs. Besides the intended target D-(UG)_12_, L-apt3.1 also binds to a few other rG4 motifs (*Kras1* rG4, *APP* rG4, *BCl2* rG4) strongly. The full gel images for the selectivity test are in Supplementary Fig. S9. (**D**) Binding between L-apt3.1 and FAM-D-(UG)_12_ mutants. No binding is detected for these three mutants.

To further explore the significance of the sequence and structure of D-apt3.1, we performed a series of mutagenesis analysis via EMSA. Various mutations were introduced at different nucleotides of D-apt3.1 (Supplementary Table S4), and their binding to L-(UG)_12_ were compared with the wild-type D-apt3.1. We designed four types of mutations: (1) base pair co-variation in stem region 2 (S2) (Fig. 3A), (2) mutations to strengthen or weaken S2 (Fig. 3B), (3) mutation of G motifs (Fig. 3C), and (4) mutations on the non-G bases in the loop and the bulge (Fig. 3D). To evaluate whether the sequence and/or base pair structure of S2 is critical for the binding to (UG)_12_, two mutants were designed, with either the first and second base pairs (apt3.1a) or the third and fourth base pairs (apt3.1b) inverted (Fig. 3A). We verified the secondary structure and ΔG of both constructs with Mfold to ensure their similarity to that of wild-type D-apt3.1. Our results showed no observable binding for both mutants, indicating that the sequence of S2 is critical for recognition, but not the base-pair structure (Fig. 3E and F). Furthermore, we designed the second mutation type to either strengthen or weaken the base pairs within S2. We introduced the GU-to-GC mutation (apt3.1c) and the GU-to-AU mutation (apt3.1d) to strengthen S2, and the GC-to-GA mutation (apt3.1e) to weaken S2 (Fig. 3B). Interestingly, we found that the binding was completely abolished upon the base pair strengthening in S2, while the binding was retained upon the base pair weakening in S2 (Fig. 3E and F). Given the fact that there are strong G4 spectroscopic signatures for D-apt3.1 (Fig. 2), it is likely that the Gs in S2 stem region may actually be involved in the rG4 structure formation instead of participating in the base pair interactions. It is also worth pointing out that the Mfold program currently cannot predict and visualize rG4 motif in their outputs. Next, we designed the third mutation type to verify the significance of the rG4 structure in D-apt3.1 for binding to (UG)_12_. We substituted the middle Gs of the GGG tracts (G_13_, G_21_) with As (apt3.1f) (Fig. 3C), and found that the mutant does not bind to L-(UG)_12_ (Fig. 3E and F). This finding further reveals the significance of rG4 structure for L-(UG)_12_ binding. Finally, we performed single nucleotide mutations on the non-G bases in the loop and bulge regions of D-apt3.1, which includes the U-to-C substitution in the loop (apt3.1g), and A-to-G substitutions in the bulge (apt3.1h, apt3.1i) (Fig. 3D). The mutants exhibited slightly weaker binding compared to the wild-type D-apt3.1, indicating that the mutation of other nucleotides in the loop and bulge region may cause local RNA conformational changes, thereby affecting binding despite not directly participating in the rG4 structure (Fig. 3E and F).

We then verified the presence of rG4 structure in each mutant using G4 ligand enhanced fluorescence spectroscopy with NMM (Supplementary Fig. S8). The intensities at a wavelength of 610 nm are higher in K^+^ condition for all four L-(UG)_12_-binding mutants (apt3.1e, apt3.1g, apt3.1h, apt3.1i), suggesting the presence of rG4 structure in these mutants. On the other hand, the rG4 formation was not observed in most of the mutants that do not bind to L-(UG)_12_, except for D-apt3.1b, demonstrating that the rG4 structure in D-apt3.1 is essential for L-(UG)_12_ recognition. The rG4 structure in D-apt3.1b is structurally distinct from that in D-apt3.1, hence even though D-apt3.1b consists of an rG4 structure, it could not recognize L-(UG)_12_. Altogether, the mutagenesis analysis showed that the rG4 structure in apt3.1 is important for (UG)_12_ recognition. We continued with D-apt3.1 for downstream studies and applications since D-apt3.1 showed the strongest binding with L-(UG)_12_.

### L-apt3.1 has a strong binding affinity and a preferential selectivity towards D-(UG)_12_ and several rG4s

To further study the binding of apt3.1 to (UG)_12_, we converted D-apt3.1 into its enantiomeric counterpart, L-apt3.1. Theoretically, L-apt3.1 should exhibit identical physical properties as D-apt3.1. To confirm this, we first conducted a series of structural analysis on L-apt3.1, including fluorescence, CD, UV melting, TDS assays and PAGE analysis. A higher fluorescence under K^+^ conditions in the presence of NMM and ThT indicates the formation of rG4 structure in L-apt3.1 (Supplementary Fig. S9A and B). The similar but inverted curve in the CD spectrum, characterized by a positive peak at 242 nm and a negative peak at 268 nm, further supports the presence of rG4 structure in L-apt3.1 (Supplementary Fig. S9C). The first derivative UV melting curve at a wavelength of 295 nm shows a decrease in absorbance under K^+^ condition, indicating the unfolding of rG4 structure in L-apt3.1 (Supplementary Fig. S9D). As observed from the hypochromic shift of the UV melting spectrum, the Tm of L-apt3.1 was determined to be 70°C, which is also the Tm of D-apt3.1, suggesting that both D- and L-apt3.1 possess an rG4 structure with identical configuration (Supplementary Fig. S9E). The similar patterns observed in the TDS assay between D-apt3.1 and L-apt3.1 support that both forms share identical physical characteristics (Supplementary Fig. S9F). Finally, the concentration-dependent UV melting spectra shows that L-apt3.1 folds into an intramolecular rG4 structure, which is also the case for D-apt3.1 (Supplementary Fig. S9G).

Additionally, we analysed the structure of the free L-apt3.1 by 1D ^1^H NMR. The NMR data are consistent with the presence of a stable helix S1 (Supplementary Fig. S10). No other stable Watson-Crick interactions are observed, consistent with mutagenesis data (Supplementary Fig. S10). The imino resonances between 10.5 and 11.5 ppm are consistent with the expected chemical shifts and hydrogen bonding interactions of guanosines in G quartets [22]. The G quartet resonances are broad due to dynamics, solvent exchange or both.

We then verified that FAM label does not affect the folding of D-(UG)_12_ using CD and UV melting assays. FAM-D-(UG)_12_ exhibits similar spectral characteristics to FAM-L-(UG)_12_, confirming the presence of the pUG fold structure (Supplementary Fig. S11). We then assessed the enantiomeric specificity of apt3.1 to (UG)_12_, and found that both D- and L-apt3.1 can only bind to (UG)_12_ of the opposite enantiomeric counterpart (Fig. 4A). These results highlight the enantiospecific nature of apt3.1 towards (UG)_12_, where the interaction depends on the chirality of the aptamer and target. Furthermore, we evaluated the binding affinity of L-apt3.1 against D-(UG)_12_ using EMSA and MST, and the Kd value was determined to be 13.5 ± 2.8 nM by EMSA and 35.9 ± 6.3 nM by MST, respectively (Fig. 4B, Supplementary Fig. S12). Notably, these Kd values are highly consistent with the Kd values for the interaction between D-apt3.1 against L-(UG)_12_ mentioned in the previous sections (Fig. 1D, Supplementary Fig. S7).

As the selection buffer consists of 1 mM MgCl_2_ and 150 mM KCl mimicking cellular ionic conditions, we studied the effect on the respective metal ions for binding of L-apt3.1 towards D-(UG)_12_. We first performed MST with varying concentrations of magnesium ions (Mg^2+^). A decrease in the Kd values was observed as the Mg^2+^ concentration increased, with a Kd value of 82.6 ± 13.7 nM for 0 mM MgCl_2_ and a Kd value of 19.8 ± 6.3 nM for 5 mM MgCl_2_, indicating that Mg^2+^ is important for the interaction of L-apt3.1 and D-(UG)_12_ (Supplementary Fig. S13). Besides, to examine the impact of K^+^ on binding, we performed MST assay with K^+^ being replaced by Li^+^, and no binding was observed (Supplementary Fig. S14), suggesting that K^+^ is critical for the stabilization of both the rG4 structures in L-apt3.1 and D-(UG)_12_.

To evaluate the selectivity of L-apt3.1 towards other structural motifs, we designed and analysed the binding of 17 other constructs via EMSA, including 5 non-G4 motifs, 4 dG4 motifs, and 8 rG4 motifs. To check whether the FAM labels affect the structural motifs, we verified the presence or absence of rG4 structures in all constructs using CD and UV melting assays. The results confirmed the presence of rG4 structures in all dG4 and rG4 motifs but not in non-G4 motifs (Supplementary Figs S15 and S16). We then assessed the binding of L-apt3.1 to non-G4 motifs including DNA and RNA hairpins, as well as single stranded homopolynucleotides (poly rA, poly rC, poly rU). No binding was observed for these five non-G4 constructs (Fig. 4C, Supplementary Fig. S17, Supplementary Table S5), suggesting that L-apt3.1 binds specifically to G4 structures and does not interact with non-G4 motifs. Likewise, no binding was observed when tested with different dG4 motifs (*BCl2Mid* dG4, *c-kit1* dG4, *c-Myc* dG4, *hTELO* dG4) (Fig. 4C, Supplementary Fig. S17, Supplementary Table S5), suggesting a preferential binding of L-apt3.1 towards rG4 motifs. Furthermore, we tested the binding of L-apt3.1 to 8 different rG4 motifs (*Kras1* rG4, *TERRA* rG4, *TRF2* rG4, *NRAS* rG4, *MT3* rG4, *APP* rG4, *BCl2* rG4, *hTERC* rG4). Compared to the intended target D-(UG)_12_, many rG4 motifs do not interact with L-apt3.1, but we also found that L-apt3.1 can recognize *APP* rG4, *BCl2* rG*4, and Kras1* rG4 (Fig. 4C, Supplementary Fig. S17, Supplementary Table S5). We further evaluated the binding affinities of these rG4 motifs using MST, and the Kd values were determined to be 36.6 ± 6.6 nM, 83.9 ± 31.1 nM, and 38.0 ± 7.8 nM for *APP* rG4, *BCl2* rG4, and *Kras1* rG4, respectively (Supplementary Fig. S18), which are similar to the binding towards (UG)_12_. The stronger binding of L-apt3.1 towards *APP* rG4, *BCl2* rG4, and *Kras1* rG4 might be due to structural similarities of these rG4 motifs as compared to the other tested rG4 motifs, where in a previous study, an *APP* rG4-targeting L-RNA aptamer binds *BCl2* rG4 and *Kras1* rG4 as well [43]. Last, we further evaluated the binding ability of (UG)_12_ by comparing it with other previously reported rG4 targeting-L-RNA aptamers [31, 36, 43, 44]. Strikingly, none of the reported L-aptamers showed significant binding to (UG)_12_ (Supplementary Fig. S19). This highlights the exceptional nature of L-apt3.1 as the first aptamer to be able to recognize pUG fold structure.

Moreover, to verify if the binding to pUG fold structure is structure specific, we designed three different (UG)_12_ mutants: (1) (UG)_12_ mut1, which consists of a U-to-C substitution (U_5_, U_11_, U_17_, U_23_) to disrupt the U quartet, (2) (UG)_12_ mut2, which consists of a G-to-A substitution (G_4_, G_10_, G_16_, G_22_) to disrupt the middle G-quartet, and (3) (TG)_12_, the DNA form of (UG)_12_. To confirm the absence of G4 structure in all three mutants, UV melting assays and NMM staining gel experiments were performed, which showed the absence of G4 structure in all three mutants, irrespective of the presence of FAM labels (Supplementary Fig. S20). We then performed EMSA on the mutants to assess their binding with L-apt3.1, and no gel shift was observed, suggesting no binding between them (Fig. 4D). The results indicate that the interaction between L-apt3.1 and (UG)_12_ is specifically dependent on the pUG fold structure. In summary, L-apt3.1 can bind strongly towards D-(UG)_12_, and a few other rG4 motifs, and its binding towards (UG)_12_ is predominantly driven by the pUG fold structure rather than the sequence.

### L-apt3.1 inhibits pUG RNA’s function in *C. elegans*

To assess the capability of L-apt3.1 *in vivo*, we evaluated the effect of L-apt3.1 treatment on gene silencing induced by pUG fold structure in *C. elegans*. Previous research demonstrated that NMM binds pUG fold structure and inhibits gene silencing [21]. We first verified the binding between D-(UG)_12_ and NMM via a microplate reader, and the Kd value was determined to be 377.4 ± 100.6 nM (Supplementary Fig. S21), which is over 15-fold higher than the Kd value between D-(UG)_12_ and L-apt3.1.

**Figure 5.**
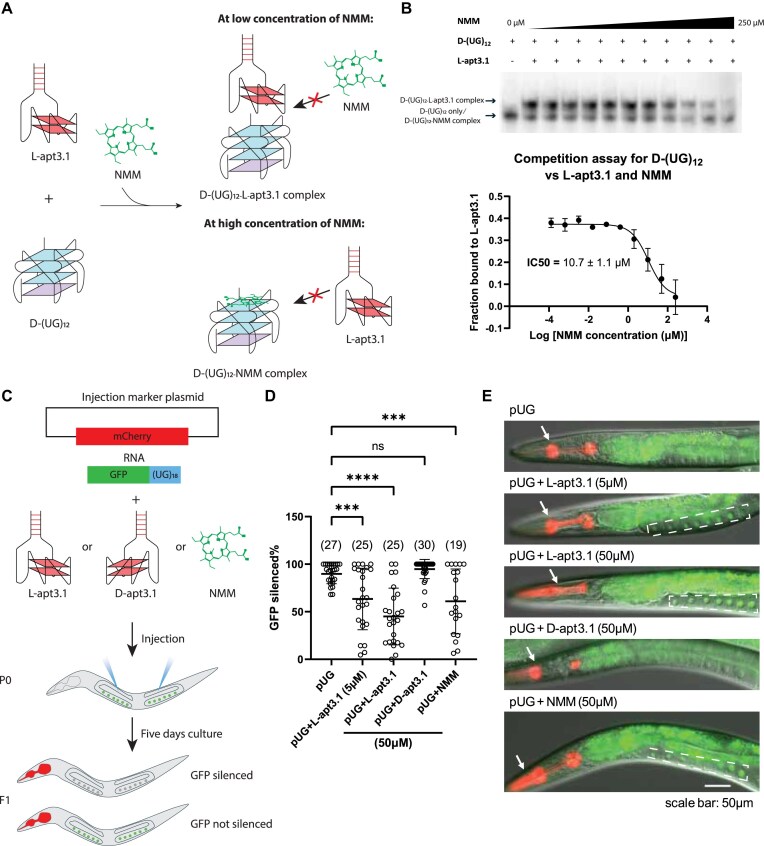
L-apt3.1 interacts strongly with D-(UG)_12_ and blocks silencing activity of pUG RNA in *C. elegans* with a stronger effect than NMM. (**A**) Schematic diagram of the competition assay for D-(UG)_12_ against L-apt3.1 and NMM. At low concentrations of NMM, the D-(UG)_12_-L-apt3.1 complex was formed, but at high concentrations of NMM, NMM displaced L-apt3.1 to form D-(UG)_12_–NMM complex instead. (**B**) The competition assay was studied using EMSA, where NMM displaced L-apt3.1 at a high concentration, with an IC50 value of 10.7 ± 1.1 μM. Data are representative of three independent experiments presented as mean ± standard deviation (SD). (**C**) Schematic diagram of the microinjection of the injection marker plasmid and GFP pUG RNA with indicated molecules (L-apt3.1, D-apt3.1 or NMM). Appearance of GFP signal indicates no inhibition of gene silencing, and no GFP signal was observed upon gene silencing. (**D**) GFP silencing efficiency of GFP pUG RNA mixed with indicated molecules. NMM served as a positive control for the inhibition of silencing activity, and L-apt3.1 showed a much greater effect for the inhibition, with a substantial effect even at a lower concentration. D-apt3.1 served as a negative control for the inhibition of silencing activity. Data are from three independent experiments presented as mean ± s.d. Each dot represents the percentage of F1 worms from individual P0s that showed silenced GFP. Numbers in parentheses are the numbers of injected P0 worms. (ns, *P* > 0.05 (*P* = 0.8650 (D-apt3.1); ***P < 0.005 (*P* = 0.0007 (5 μM L-apt3.1) and *P* = 0.0005 (50 μM NMM); ****P < 0.0005 (*P* < 0.0001 (50 μM L-apt3.1), ns represents not significant) (one-way ANOVA with Tukey’s post-hoc tests). (**E**) Representative images showing GFP expression patterns after pUG injection with indicated molecules observed under 20 × lens. Worms expressing the injection marker plasmid (*myo-2p::mCherry*) showed red fluorescence in the pharynxes as denoted with the white arrows, indicating successful incorporation of injected materials in these worms. Only injection marker positive worms were used for GFP expression evaluation. If not silenced, GFP is expressed in the nuclei of oocytes, showing as distinct arrays of dots. Dashed polygons mark the areas to check GFP expression.

To directly compare the binding of D-(UG)_12_ against L-apt3.1 and NMM, we tested whether NMM can compete with L-apt3.1 to bind D-(UG)_12_ using EMSA (Fig. 5A). By adding 250 nM L-apt3.1 and varying concentrations of NMM to D-(UG)_12_ simultaneously, NMM was found to interact with D-(UG)_12_, with a half maximal inhibitory concentration (IC50) of 10.7 ± 1.1 μM (Fig. 5B), supporting that L-apt3.1 binds to D-(UG)_12_ much stronger than NMM. Also, based on the X-ray crystallography, NMM interacts with pUG fold by stacking on the top G-quartet in pUG fold [21]. As NMM displaced L-apt3.1 from the D-(UG)_12_-L- apt3.1 complex at high concentrations, we hypothesized that L-apt3.1 and NMM share the same binding site, where L-apt3.1 potentially interacts, either completely or partially, with the G-quartet in the pUG fold structure. To further verify this, we evaluated the ability of L-apt3.1 to displace NMM from D-(UG)_12_–NMM complex. Remarkably, even at low concentrations of L-apt3.1, we observed the formation of D-(UG)_12_-L-apt3.1 complex (Supplementary Fig. S22), suggesting that D-(UG)_12_ preferably binds to L-apt3.1 over NMM. However, as the band shift upon NMM binding to D-(UG)_12_ is not clear, it is challenging to distinguish between D-(UG)_12_ only and D-(UG)_12_-NMM complex, hence the IC50 value of L-apt3.1 could not be determined accurately and thus not reported in this study.

L-RNA, the enantiomer of the naturally occurring D-RNA, could not be recognized by nuclease due to its distinct stereochemistry. To evaluate the stability of L-apt3.1 in a complex biological milieu, we conducted a 2-h stability test in 5% fetal bovine serum (FBS) using denaturing gel electrophoresis. Notably, no degradation of L-apt3.1 was observed throughout the experiment (Supplementary Fig. S23), suggesting its robust stability in biological environments, making it suitable for *in vivo* studies.

*In vitro* transcribed RNA containing pUG tails silences *oma-1* gene expression when injected into the syncytial germline of *C. elegans* [23]. This pUG RNA-mediated gene silencing requires the pUG fold structure, which recruits RdRP for the synthesis of gene silencing siRNAs. NMM can inhibit the silencing effect by blocking the recruitment of RdRP by pUG fold [21]. We asked whether L-apt3.1 could also block pUG RNA-mediated gene silencing *in vivo*, and to further compare the effect with NMM (Fig. 5C, Supplementary Fig. S24). We first pre-treated GFP pUG RNA with either buffer alone (untreated control), NMM (positive control), D-apt3.1 (negative control), or L-apt3.1. We then injected the pUG RNA mix into P0 worms with GFP expressed in the germline together with an injection marker plasmid to mark F1 worms that successfully inherit the injected materials. GFP pUG RNA-induced silencing of GFP was evaluated in F1 worms (Fig. 5C). While pUG RNA alone induced efficient GFP silencing, pUG RNA injected together with 50 μM L-apt3.1 or NMM substantially compromised gene silencing potency, with L-apt3.1 showing a more dramatic inhibition (*P* < 0.0005 for 50 μM L-apt3.1; *P* = 0.0005 for 50 μM NMM/mean difference = 44.8 ± 6.3 for 50 μM L-apt3.1; mean difference = 29.0 ± 6.3 for 50 μM NMM, compared to pUG only) (Fig. 5D and E, Supplementary Fig. S25). However, GFP pUG RNA injected together with 50 μM D-apt3.1 showed no difference in silencing GFP expression (*P* = 0.8650 / mean difference = -5.0 ± 5.6), confirming the specificity of L-apt3.1 (Fig. 5D and E, Supplementary Fig. S25). Given that L-apt3.1 has a much higher affinity to pUG fold than NMM does, we tested a lower concentration of L-apt3.1 (5 μM), which also showed significant inhibition of GFP silencing (*P* = 0.0007 / mean difference = 26.5 ± 5.8).

We next assessed the inhibitory effects of the antisense oligonucleotide (ASO) on gene silencing induced by pUG fold. As previous, we pre-treated GFP pUG RNA with buffer only and (CA)_18_, including without pre-annealing and with pre-annealing with GFP pUG RNA. Surprisingly, (CA)_18_ can lead to almost complete inhibition of the gene silencing regardless whether it is pre-annealed with pUG RNA (*P* < 0.0005 for not pre-annealed (CA)_18_; *P* < 0.0005 for pre-annealed (CA)_18_ / mean difference = 86.3 ± 6.1 for not pre-annealed (CA)_18_; mean difference = 87.3 ± 5.8 for pre-annealed (CA)_18_, compared to pUG only) (Supplementary Fig. S26). We deduce that this might be due to the unwinding of pUG fold by (AC)_18_, resulting in the complete disruption of the pUG fold structure.

Finally, we examined the toxicity of different molecules, including L-apt3.1, D-apt3.1, NMM, and (CA)_18_, in *C. elegans* by assessing the impact on the brood size of the germlines. Overall, as compared to the control group, no significant reduction in the brood size was observed (*P* = 0.8991 for L-apt3.1; *P* = 0.5247 for D-apt3.1; *P* = 0.8749 for NMM; *P* = 0.9990 for not pre-annealed (CA)_18_; *P* > 0.9999 for pre-annealed (CA)_18_) (Supplementary Fig. S27), suggesting that the toxicity towards *C. elegans* is minimal or negligible in our experimental conditions. Collectively, while both L-apt3.1 and NMM can bind pUG fold structure and inhibit gene silencing induced by pUG fold structure, L-apt3.1 exhibited more robust efficacy than NMM *in vitro* and *in vivo*.

## Discussion

Targeting G4 structures has been important to explore their roles in various cellular processes [45–47]. To date, several L-RNA aptamers recognizing different G4 motifs have been reported, demonstrating their potential in regulating G4-mediated biological activities, including transcription and translation [31, 36, 43, 44]. The remarkable nuclease resistance and biostability of L-RNA aptamer make it a promising candidate for targeting G4 structures *in vivo*. In line with this, we reported a novel L-RNA aptamer for the recognition of pUG fold structure, a unique rG4 motif recently discovered [21, 22]. We employed G4-SELEX-seq for the identification of pUG fold-binding aptamer, and successfully obtained D-apt3.1 as the L-(UG)_12_ binding aptamer, with a hairpin structure and a bulge predicted by Mfold. Interestingly, further structural characterization of D-apt3.1 revealed the presence of a parallel rG4 motif, which contradicted with the predicted secondary structure. This rG4 structure was failed to be predicted by Mfold as the program does not consider G4 motif, underscoring the need to verify the secondary structure in aptamer candidate, especially when the sequence is G-rich. In this case, computational G4 prediction methods can offer initial insights into the potential presence of G4 structure; however, the results are not always accurate. The G4H and G4NN scores presented conflicting results for the rG4 formation in D-apt3.1, emphasizing the importance to confirm the G4 structure experimentally. Further investigation on D-apt3.1 reveals that it forms an intramolecular G4 structure. It is of note that another study have shown that G4-containing aptamers potentially form multimers through intermolecular interactions [48]. Understanding the dynamics of both intra- and intermolecular G4 structures in aptamers could provide insights into their functional roles in biological activities. The rG4 motif in D-apt3.1 was further demonstrated to play a key role in the interaction with pUG fold by mutagenesis analysis. This is not unexpected as we also previously observed and reported the presence of rG4 structural motif in other G4-targeting L-RNA aptamers [31, 36, 43, 44]. However, since pUG fold is structurally distinct from other known G4 structures and adopts a left-handed structure, the interaction between pUG fold and the aptamer should be quite different from other G4-aptamer interactions. We believe that three-dimensional structural complex determination and investigation using X-ray crystallography and NMR can likely provide a more comprehensive understanding on the structural basis of pUG fold-L- apt3.1 interaction. A deeper knowledge of the interaction can also provide valuable insights for further optimization of L-apt3.1 to improve the binding performance.

L-apt3.1 demonstrated a strong binding affinity towards pUG fold, reaching a nanomolar range. To the best of our knowledge, L-apt3.1 represents the first L-RNA aptamer capable of recognizing pUG fold structure. In this study, we evaluated the binding of pUG fold to previously reported G4-targeting L-RNA aptamer, of which none of them bind to pUG fold. The lack of binding between pUG fold and the general G4-targeting L-RNA aptamers, including L-apt4-1c [31] and L-Apt12-6 [36], is unexpected as these aptamers are known to recognize different G4 motifs. We hypothesized that the distinct conformational characteristics of pUG fold may contribute to the lack of binding observed with these aptamers. These aptamers might recognize structural features that do not exist in pUG fold, such as the right-handed backbone or the consecutive Gs in the G-tracts. However, although L-apt3.1 demonstrates substantial selectivity towards rG4s over dG4s and non-G4s, it could also bind to a few other rG4 motifs reported in human, including *APP* rG4, *Kras1* rG4, and *BCl2* rG4. We hypothesize that L-apt3.1 primarily recognizes the G-quartet, which contributes to the off-target binding effects. This hypothesis is further supported by the results of the NMM competition assay, which demonstrates that L-apt3.1 and NMM can compete for binding to pUG fold. Moreover, given that the binding affinity varies across different rG4 motifs, it is likely that L-apt3.1 interacts with other unique features of pUG fold besides the G-quartet. Overall, these findings demonstrate promising selectivity of L-apt3.1 towards non-rG4 motifs, however, it shows preferences for binding to rG4 motifs besides pUG fold. L-apt3.1 still has room for improvement for its selectivity and requires additional efforts to achieve individual rG4 selectivity. The high degree of structural resemblance among various G4 constructs, characterized by features like stacking of G-quartets and G-tract runs, is a major challenge in achieving precise targeting. Detailed structural analysis of specific G4 motifs is essential to identify unique features that differentiate them from other G4 motifs. These distinctive characteristics could serve as the basis for targeting in selection processes. Modified SELEX is a potential approach to enhance the selectivity of G4-targeting aptamers by focusing on the distinct features of specific G4 motif. Integration of additional strategies to SELEX, such as refined library template design or additional tailored selection steps, has the potential to enhance the recognition of the unique features of the G4 target. Further research and optimization efforts in this direction are underway in the laboratory, and it is critical to develop a high selectivity aptamer as a targeting tool for further exploration of the biological significance of pUG fold.

To gain more insights on the potential applications of L-apt3.1, we investigated the regulation of gene expression by L-apt3.1 *in vivo*. We demonstrate that L-apt3.1 effectively inhibits pUG fold-mediated gene silencing activity in *C. elegans*. This mechanism likely involves the binding of L-apt3.1 to pUG fold, which prevents the recruitment of RdRP for siRNA synthesis. A previous study presented evidence that NMM binds the pUG fold to inhibit gene silencing activity in *C. elegans* [21]. Nevertheless, the binding affinity and selectivity of NMM remain a big issue. The binding between NMM and pUG fold is not very strong as compared to L-apt3.1, and NMM can bind numerous parallel dG4 and rG4 motifs [24, 25]. This poses a big challenge for NMM to efficiently target pUG fold *in vivo*, particularly in living systems with diverse G4 motifs. In contrast, the stronger binding of L-apt3.1 towards pUG fold, and the enhanced selectivity of L-apt3.1 over NMM, make L-apt3.1 a much better candidate to modulate gene expression associated with pUG fold. Herein, our findings show that L-apt3.1 exhibits a comparable performance as NMM in inhibiting gene silencing. Interestingly, even at significantly lower concentrations, L-apt3.1 achieves a comparable effect to NMM. This enhanced efficacy of L-apt3.1 in regulating the gene expression is likely attributed to its stronger binding affinity with pUG fold. This finding suggests that L-apt3.1 can inhibit gene regulatory pathways associated with the pUG fold in *C. elegans*, and also potentially in other living systems such as human.

On the other hand, while (AC)_18_ possesses a more robust efficiency on the gene silencing inhibition, it is essential to note the distinct mechanisms between ASOs and aptamers. ASOs target specific DNA or RNA sequences, while aptamers target specific structural motifs. In the context of pUG fold-targeting, ASOs recognize the pUG sequences and unwind pUG fold structures, whereas aptamers typically target and stabilize the pUG fold structures. Also, while there are numerous UG repeats present in living systems, not all are long enough to fold into pUG fold structures (>11.5 GU repeats). ASOs lack the ability to differentiate between pUG sequences and pUG fold structures, and they likely prefer the unfolded pUG sequence over the folded pUG fold structure. While they serve as valuable tools for inhibiting specific cellular activities mediated by G4 targets, it is challenging for ASOs in direct targeting of pUG fold structure. In contrast, aptamers offer advantages for studying the biological roles of pUG fold structures, providing a comprehensive approach to investigate the complex biology associated with pUG fold. Nevertheless, ASOs might be a potential strategy to improve the binding affinity and selectivity of aptamers. Recent studies have demonstrated that conjugation between rG4-targeting aptamers and ASOs complementary to flanking sequences can facilitate the aptamer-rG4 target interactions while minimizing off-target effects [49, 50]. This approach might be particularly beneficial for targeting specific pUG fold structures in cellular or *in vivo* environments. By leveraging both ASO and aptamer technologies, we can possibly expand our understanding of gene regulations related to specific rG4 motifs, highlighting the potentials for targeted therapeutic strategies aimed at individual rG4 structures.

To date, over 20 000 pUG fold sequences have been predicted within the human transcriptome [21]. Nonetheless, since RdRPs are not found in human [21], the biological function of pUG fold is yet to be studied. Lately, Jansson-Fritzberg et al. discovered that pUG fold can bind and inhibit the activity of DNA methyltransferase (DNMT1) protein *in vitro* with higher affinity compared to canonical rG4 structures [51]. This finding suggests that pUG fold may indeed play important biological roles in human. Given the robust efficacy in inhibiting gene silencing in *C. elegans*, L-apt3.1 is a promising candidate for exploration of pUG fold biology in human in the future.

## Conclusion

In this original work, we present L-apt3.1 as a novel L-RNA aptamer interacting with pUG fold structure using G4-SELEX-seq. Structural analysis reveals a parallel rG4 motif within L-apt3.1 important for pUG fold recognition. The interaction between L-apt3.1 and pUG fold is strong in nanomolar affinity, with a high dependence towards chirality. While L-apt3.1 recognizes a few other rG4 motifs in human, we showcase that L-apt3.1 is the first L-RNA aptamer able to bind pUG fold specifically. We also show that L-apt3.1 exhibits excellent biostability in cellular environment and minimal toxicity *in vivo*, making it highly suitable for both in cell and *in vivo* applications. Notably, we demonstrate the high gene-silencing inhibition efficiency of L-apt3.1 *in vivo* in *C. elegans*, highlighting the advantages of using L-RNA aptamers for controlling G4-linked functions. This work demonstrates the first use of L-RNA aptamer for *in vivo* G4-mediated gene regulation, making a significant breakthrough in the field of RNA-based gene control. We envision L-apt3.1 as a potential tool for studying pUG fold structure within living systems. Further development and optimization of L-RNA aptamers can likely unlock their capabilities for diverse chemical and biological applications.

## Supplementary Material

gkaf137_Supplemental_File

## Data Availability

The data underlying this article are available in the article and in its online supplementary material. Further data will be shared on reasonable request to the corresponding author.
