# Supplementary material for: A novel L-RNA aptamer to regulate the pUG fold RNA-induced gene expression in vivo
Source: Nucleic Acids Res. 2025 Mar 8;53(5):gkaf137. doi: 10.1093/nar/gkaf137 (PMC11890061; doi:10.1093/nar/gkaf137)
Supplement: gkaf137_Supplemental_File [file gkaf137_supplemental_file.pdf]

Supplementary Information

A novel L-RNA aptamer to regulate the pUG fold RNA-induced gene expression *in vivo*

Shiau Wei Liew[1], Dong Cao[2], Riley J. Petersen[3], Samuel E. Butcher[3], Scott G. Kennedy[2], Chun Kit Kwok[1,4,*]

[1] Department of Chemistry and State Key Laboratory of Marine Pollution, City University of Hong Kong, Kowloon Tong, Hong Kong SAR, China

[2] Department of Genetics, Blavatnik Institute at Harvard Medical School, Boston, MA 02115, USA

[3] Department of Biochemistry, University of Wisconsin-Madison, Madison, WI 53706, USA

[4] Shenzhen Research Institute of City University of Hong Kong, Shenzhen, China

*Corresponding author. Email: ckkwok42@cityu.edu.hk

Table S1. Oligonucleotides used in this study.

Table S2. Conditions used and number of PCR cycles throughout the selection process.

Table S3. Sequences of aptamer candidates selected.

Table S4. Sequences of apt3.1 mutants.

Table S5. Binding of L-apt3.1 towards different structural motifs.

Figure S1. Spectroscopic analysis of D-$(UG)_{12}$ verifies the folding of the pUG fold structure.

Figure S2. Spectroscopic analysis of L-$(UG)_{12}$ confirms the folding of pUG fold structure in the opposite chirality.

Figure S3. The Mfold predicted secondary structures of aptamer candidates with and without fixed linker region.

Figure S4. Spectroscopic analysis of FAM-L-$(UG)_{12}$ verifies the formation of the pUG fold structure.

Figure S5. Initial binding test of aptamer candidates versus FAM-L-$(UG)_{12}$.

Figure S6. The binding between FAM-L-$(UG)_{12}$ and the aptamer candidates with stronger binding via EMSA.

Figure S7. The binding of FAM-L-$(UG)_{12}$ against D-apt3 and D-apt3.1 via MST.

Figure S8. NMM enhanced fluorescence spectroscopy validates the presence of rG4 structure in D-apt3.1 mutants.

Figure S9. Structural analysis of L-apt3.1 shows that L-apt3.1 shares an identical configuration with opposite chirality as D-apt3.1.

Figure S10. 1D NMR analysis of L-apt3.1.

Figure S11. Spectroscopic analysis of FAM-D-$(UG)_{12}$ reveals that the FAM label does not affect the pUG fold structure folding.

Figure S12. The binding between FAM-D-$(UG)_{12}$ and L-apt3.1 via MST.

Figure S13. $MgCl_2$ dependency test for the binding between FAM-D-$(UG)_{12}$ and L-apt3.1 via MST.

Figure S14. The binding between FAM-D-$(UG)_{12}$ and L-apt3.1 under Li^+ condition via MST.

Figure S15. CD spectra to verify the presence or absence of G4 structure in the FAM-labelled off-target structural motifs.

Figure S16. UV melting spectra to verify the presence or absence of G4 structure in the FAM-labelled off-target structural motifs.

Figure S17. Unsliced gel for the selectivity test for L-apt3.1 against other FAM-labelled structural constructs.

Figure S18. The binding between L-apt3.1 and 3 other FAM-labelled rG4 motifs via MST.

Figure S19. The binding between FAM-D-$(UG)_{12}$ and other known rG4-targeting L-RNA aptamers via EMSA.

Figure S20. Spectroscopic analysis of D-$(UG)_{12}$ mutants to evaluate the presence of G4 structures.

Figure S21. The binding between D-$(UG)_{12}$ and NMM via microplate reader.

Figure S22. Displacement assay shows L-apt3.1 competing with NMM to bind with D-$(UG)_{12}$.

Figure S23. Stability test of L-apt3.1 demonstrates the excellent stability of L-apt3.1 in biological milieu.

Figure S24. Schematic diagram of the mechanism of gene silencing inhibition by L-apt3.1.

Figure S25. GFP expression in *C. elegans* for the study of gene silencing inhibition of indicated molecules.

Figure S26. GFP silencing efficiency of GFP pUG RNA mixed with $(CA)_{18}$.

Figure S27. Brood size assay reveals that all injected molecules exhibited a negligible toxicity effect towards *C. elegans*.

Table S1. Oligonucleotides used in this study.

Name	Sequences (5' to 3')	Length
DNA N30 nor library	TTCTAATACGACTCACTATAGGTTACCAGCCTTCACTGC (N30) GCACCACGGTCGGTCACAC<br>N = A:T:C:G = 25:25:25:25	88
DNA N30 30% GC library	TTCTAATACGACTCACTATAGGTTACCAGCCTTCACTGC (N30) GCACCACGGTCGGTCACAC<br>N = A:T:C:G = 35:35:15:15	88
Forward primer	TTCTAATACGACTCACTATAGGTTACCAGCCTTCACTGC	39
Reverse primer	GTGTGACCGACCGTGGTGC	19
Biotin-L-$(UG)_{12}$	Biotin-L-UGUGUGUGUGUGUGUGUGUGUGUG	24
FAM-L-$(UG)_{12}$	FAM-L-UGUGUGUGUGUGUGUGUGUGUGUG	24
FAM-D-$(UG)_{12}$	FAM-UGUGUGUGUGUGUGUGUGUGUGUG	24
FAM-D-*Kras1* rG4	FAM-GCGGCGGCGGAGGCA	15
FAM-D-*TERRA* rG4	FAM-UUAGGGUUAGGGUUAGGGUUAGGG	24
FAM-D-*TRF2* rG4	FAM-CGGGAGGGCGGGGAGGGC	18
FAM-D-*MT3* rG4	FAM-GAGGGAGGGAGGGAGAGGGA	20
FAM-D-*NRAS* rG4	FAM-GGGAGGGGCGGGUCUGGG	18
FAM-D-*APP* rG4	FAM-CGGGGCGGGUGGGGAGGGGU	20
FAM-D-*Bcl2* rG4	FAM-GGGGGCCGUGGGGUGGGAGCUGGGG	25
FAM-D-*hTERC* rG4	FAM-GGGUUGCGGAGGGUGGGCCU	20

FAM-D-*BCl2Mid* dG4	FAM-GGGCGCGGGAGGAAGGGGGCGGG	23
FAM-D-*cKit1* dG4	FAM-AGGGAGGGCGCTGGGAGGAGGG	22
FAM-D-*c-Myc* dG4	FAM-TGAGGGTGGGTAGGGTGGGTAA	22
FAM D-*hTELO* dG4	FAM-TTAGGGTTAGGGTTAGGGTTAGGG	24
FAM-D-poly rA	FAM-AAAAAAAAAAAAAAAAAA	18
FAM-D-poly rC	FAM-CCCCCCCCCCCCCCCCCC	18
FAM-D-poly rU	FAM-UUUUUUUUUUUUUUUUUU	18
FAM-D-RNA hairpin	FAM-CAGUACAGAUCUGUACUG	18
FAM-D-DNA hairpin	FAM-CAGTACAGATCTGTACTG	18
L-apt4-1c	GCCCUAAAGGUGGUGGUGGGAGGGC	25
L-Ap3-7	GGCCUCACGGCGGGUGGGUGGGUUAGCUCGAUGGCC	36
L-Apt.8f	GCGGCAAGAGUGUGGGAGGGGGUCGACGCCGC	33
L-Apt12-6	CGCCGCCGGGUAUGAGGGAGGAGGGGGCGGCG	32
FAM-D-$(UG)_{12}$ mut1*	FAM-UGU**A**UGUGU**A**UGUGU**A**UGUGU**A**UG	24
FAM-D-$(UG)_{12}$ mut2*	FAM-UGUG**C**GUGUG**C**GUGUG**C**GUGUG**C**G	24
FAM-D-$(TG)_{12}$	FAM-TGTGTGTGTGTGTGTGTGTGTGTG	24
T7 DNA template for GFP-$(UG)_{18}$[#]	TAATACGACTCACTATAGGGAGACCACATGAGTAAAGGAGAAGAACTTT TCACTGGAGTTGTCCCAATTCTTGTTGAATTAGATGGTGATGTTAATGG GCACAAATTTTCTGTCAGTGGAGAGGGTGAAGGTGATGCAACATACGGA AAACTTACCCTTAAATTTATTTGCACTACTGGAAAACTACCTGTTCCAT GGCCAACACTTGTCACTACTTTCTGTTATGGTGTTCAATGCTTCTCGAG	432

	ATACCCAGATCATATGAAACGGCATGACTTTTTCAAGAGTGCCATGCCC<br>GAAGGTTATGTACAGGAAAGAACTATATTTTTCAAAGATGACGGGAACT<br>ACAAGACACGTGCTGAAGTCAAGTTTGAAGGTGATACCCTTGTTAATAG<br>AATCgtgtgtgtgtgtgtgtgtgtgtgtgtgtgtgtgt	

* The mutated nucleotides are denoted in blue.

# The T7 promoter site is indicated with an underline and the $(UG)_{18}$ sequence is represented with the lowercase letters.

Table S2. Conditions used and number of PCR cycles throughout the selection process.

**Rounds**	**1**		**2**		**3**		**4**		**5**		**6**		**7**	
$MgCl_2$ Conc. (mM)	5		5		5		5		1		1		1	
D-RNA pool (pmol)	1000		100		45		30		30		15		6	
Target L-pUG fold (pmol)	200		200		200		100		30		15		6	
Negative selection (h)	2		2		2		1		1		1		1	
Positive selection (min)	30		30		30		30		30		30		30	
Washing (min)	1*		1*		1*		10		10		10		10	
Temperature (°C)	25		25		37		37		37		37		37	
PCR cycles[#]	11	11	9	11	8	13	7	9	10	11	8	9	9	10

* Pipette mixing was performed for the washing step.

[#] The PCR cycles number are for N30 nor library and N30 30% GC library in each round, respectively.

Table S3. Sequences of aptamer candidates selected.

Name	Sequence (5' to 3')	G4H*	G4NN*	Library
**apt1**	GUACUGGGGGUGGGCGGCGGGGAAACAGAC	1.5333	0.9277	N30 nor
**apt2**	GGUGUUGGGGUUCACGGAGGGAGGGACGCC	1.2333	0.9597	
**apt3**	GGGGCACUGGUGGGUCGGUGGGAAGCCCCA	0.8000	0.5270	
**apt4**	GGUCGAACUGGGCAUGGGAUUGUUCGAGCU	0.7000	0.4191	
**apt5**	AUGCAACGGGGUAAACGGUGGGAGGGACAU	1.1667	0.9684	N30 30% GC
**apt6**	AUUAAAUACUGGGCAUGGGAUUGUAUUGAU	0.6000	0.1788	
**apt7**	AUGAUAGGGUACAUGGUGGGAUGGAUUCAU	0.8333	0.8400	
**apt8**	UGUGAUACGGGUGGGAUAAGGUGGGUCAUA	1.0333	0.9750	
**apt9**	ACGUAUAUCUGGGCAUGGGAUUGAUAUAUU	0.5667	0.0091	

* The prediction scores above threshold are denoted in green.

Table S4. Sequences of apt3.1 mutants.

Name	Sequence (5' to 3')*	G4H#	G4NN#	Type
**apt3.1**	GCCGCACUGGUGGGUCGGUGGGAAGCGGC	0.8276	0.7407	WT
**apt3.1a**	GCCGCA**GG**GGUGGGUC**CU**UGGGAAGCGGC	1.0345	0.7869	co-variation mutation in S2
**apt3.1b**	GCCGCACU**UC**UGGG**GG**GGUGGGAAGCGGC	1.2069	0.9431	
**apt3.1c**	GCCGCAC**C**GGUGGG**C**CGGUGGGAAGCGGC	0.6207	0.6378	Strengthen S2
**apt3.1d**	GCCGCACUG**A**UGGGUC**A**GUGGGAAGCGGC	0.6207	0.5908	
**apt3.1e**	GCCGCA**A**UGGUGGGU**A**GGUGGGAAGCGGC	0.8966	0.8245	Weaken S2
**apt3.1f**	GCCGCACUGGUG**A**GUCGGUG**A**GAAGCGGC	0.3448	0.1238	G motifs
**apt3.1g**	GCCGCACUGG**C**GGGUCGGUGGGAAGCGGC	0.7931	0.6911	non-G bases in loop or bulge
**apt3.1h**	GCCGCACUGGUGGGUCGGUGGG**G**AGCGGC	1.0690	0.8143	
**apt3.1i**	GCCGCACUGGUGGGUCGGUGGGA**G**GCGGC	0.9310	0.9310	

* The mutated nucleotides with respect to apt3.1 are denoted in purple.
# The prediction scores above threshold are denoted in green.

Table S5. Binding of L-apt3.1 towards different structural motifs.

Name	Structural motifs	Binding to L-apt3.1
$(UG)_{12}$	rG4	Strong binding
Kras1 rG4		Strong binding
APP rG4		Strong binding
BCl2 rG4		Strong binding
TRF2 rG4		Weak binding
NRAS rG4		No binding
TERRA rG4		No binding
MT3 rG4		No binding
hTERC rG4		N binding
BCl2Mid dG4	dG4	No binding
c-Kit1 dG4		No binding
c-Myc dG4		No binding
hTELO dG4		No binding
Poly rA	Non-G4	No binding
Poly rC		No binding
Poly rU		No binding
RNA hairpin		No binding
DNA hairpin		No binding

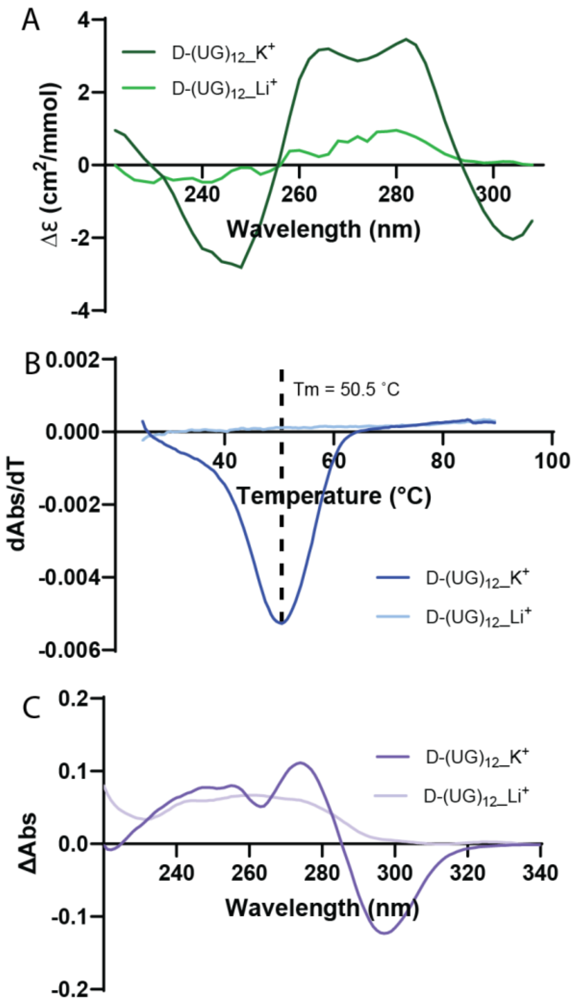

Figure S1. Spectroscopic analysis of D-(UG)$_{12}$ verifies the folding of the pUG fold structure. (A) CD spectrum. A negative peak at 250 nm and a positive doublet at 265 nm and 285 nm were observed, indicating the formation of pUG fold structure. (B) UV melting spectrum. A hypochromic shift was observed with a maximum negative value observed at 295nm, suggesting the formation of rG4 structure. The melting temperature (Tm) was determined to be 50.5 °C. (C) TDS spectrum. The presence of G4 structure in the pUG fold sequence is indicated by two positive peaks at 247 nm and 274 nm and a negative peak at 297 nm.

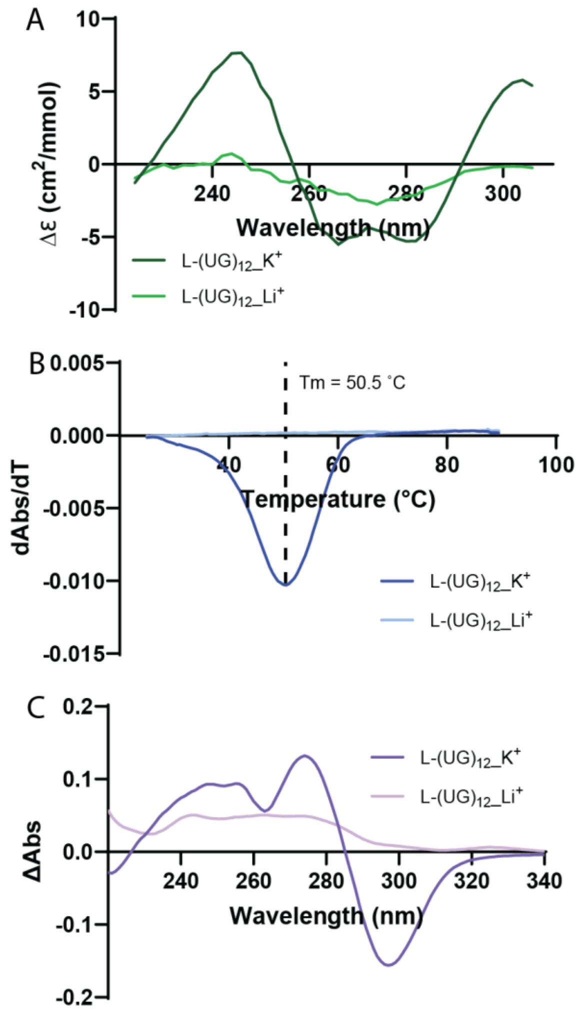

Figure S2. Spectroscopic analysis of L-$(UG)_{12}$ confirms the folding of pUG fold structure in the opposite chirality. (A) CD spectrum. The inverted spectrum from D-$(UG)_{12}$, characterized with a positive peak at 246 nm and a negative doublet at 266 nm and 280 nm, verified the presence of L-form pUG fold structure. (B) UV melting spectrum. The maximum negative value of the hypochromic shift indicates the formation of rG4 structure with a Tm of 50.5 °C, which is the same as D-$(UG)_{12}$. (C) TDS spectrum. The spectrum has a similar pattern as that of D-$(GU)_{12}$, with positive peaks at 248 nm and 274 nm and the negative peak at 297 nm, suggesting the presence of an rG4 structure with an identical configuration as D-$(UG)_{12}$.

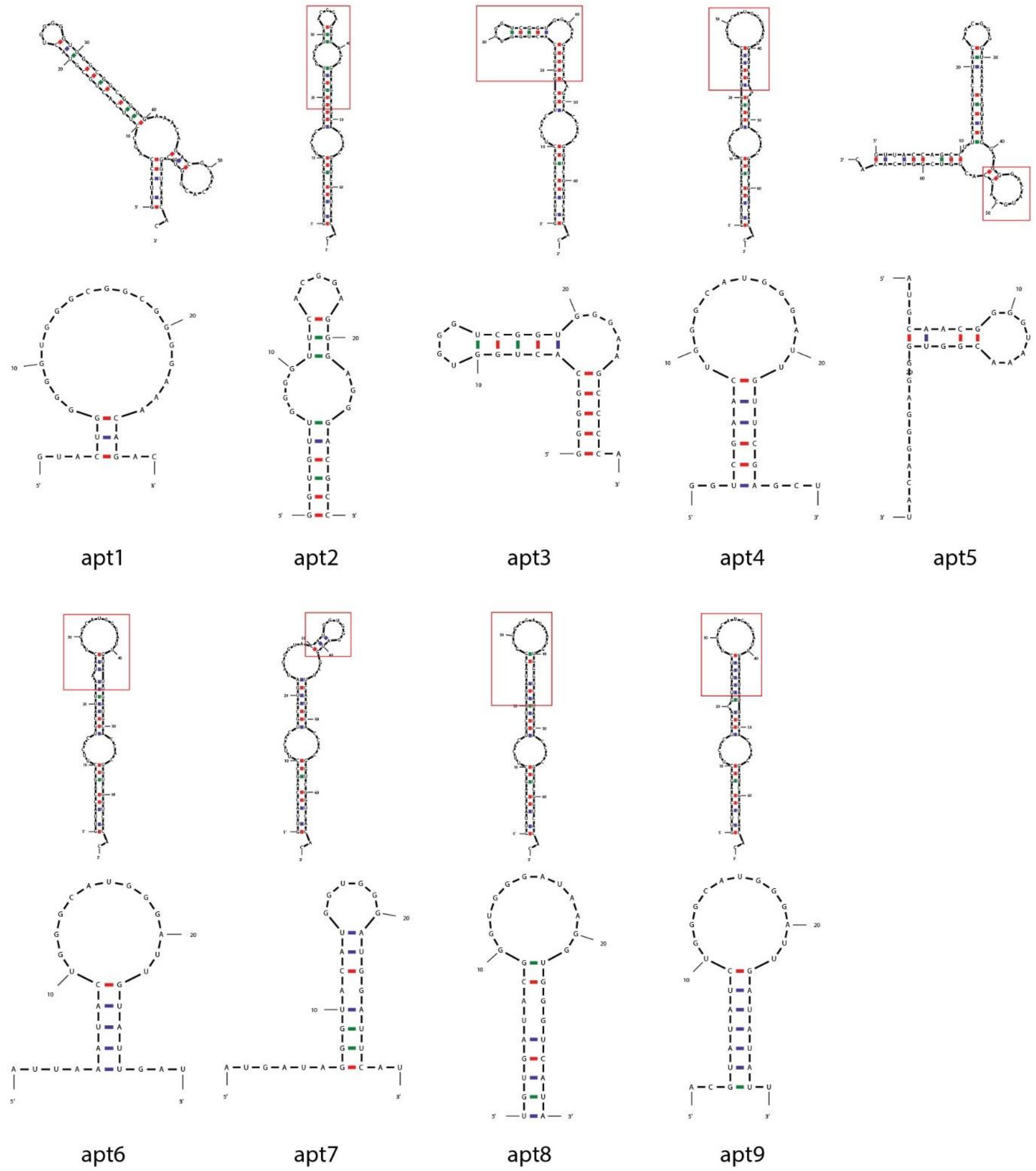

Figure S3. The Mfold predicted secondary structures of aptamer candidates with and without fixed linker region. The red boxed regions refer to the structures that are found in both with and without linker sequences. Apt3 was selected for downstream analysis with the optimized aptamer shown in Figure 1C.

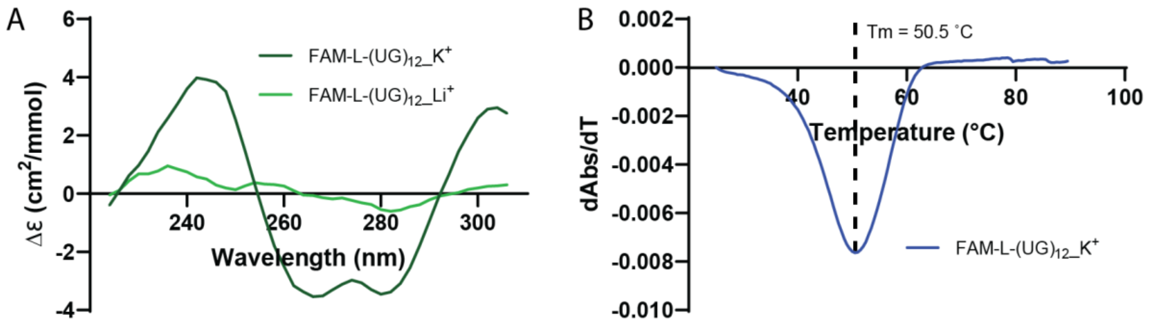

Figure S4. Spectroscopic analysis of FAM-L-$(UG)_{12}$ verifies the formation of the pUG fold structure. (A) CD spectrum. The similar trend with L-$(UG)_{12}$, with a positive peak at 242 nm and negative peaks at 266 nm and 280 nm, illustrates that the FAM label does not interfere with the pUG fold structure. (B) UV melting spectrum. The Tm at 50.5 °C is the same with L-$(UG)_{12}$, further supporting that the FAM label does not affect the folding of pUG fold structure.

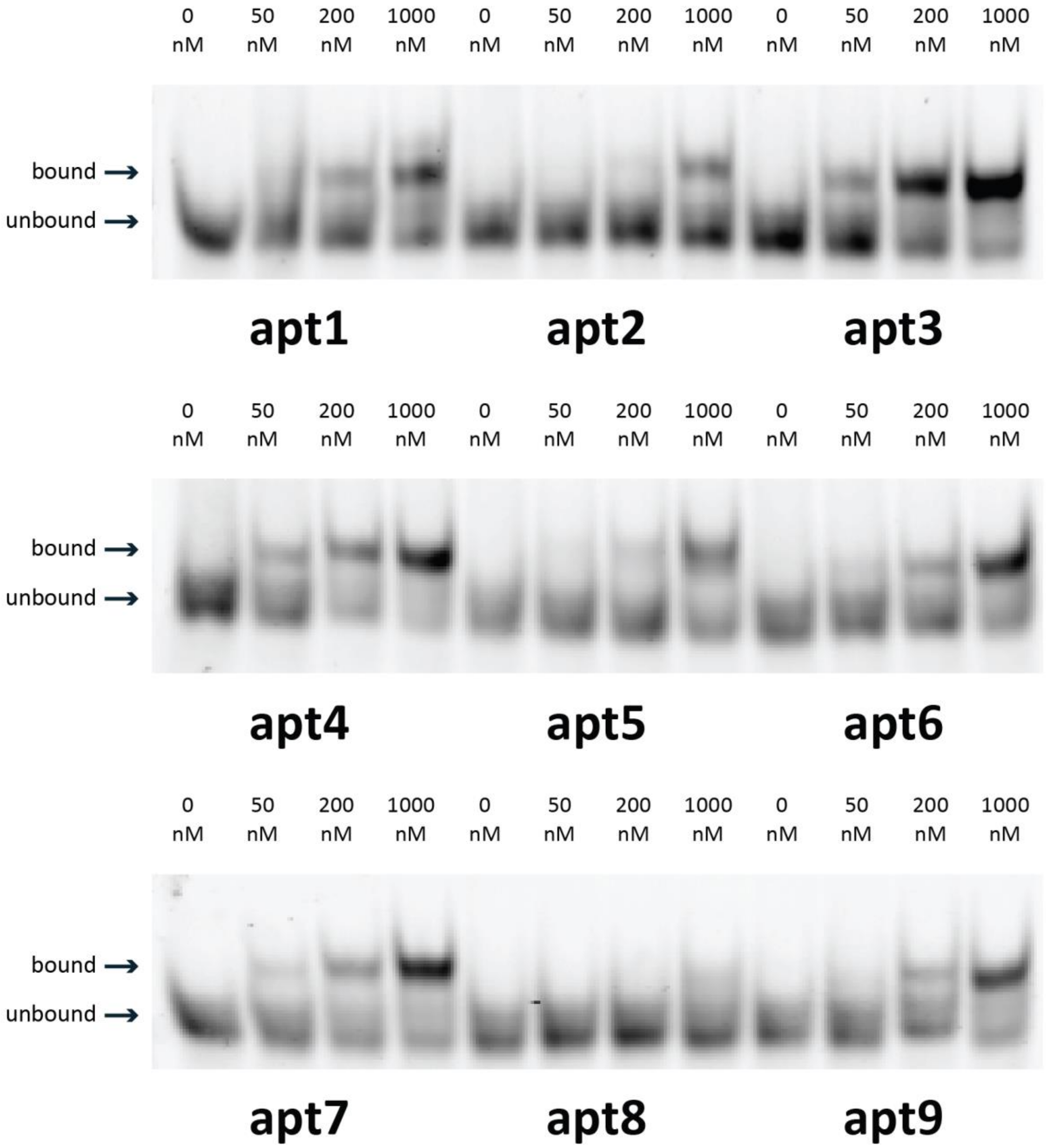

Figure S5. Initial binding test of aptamer candidates versus FAM-L-$(UG)_{12}$. The binding between the L-pUG fold and aptamer candidates was strongest for apt3, hence apt3 was selected for the downstream experiments.

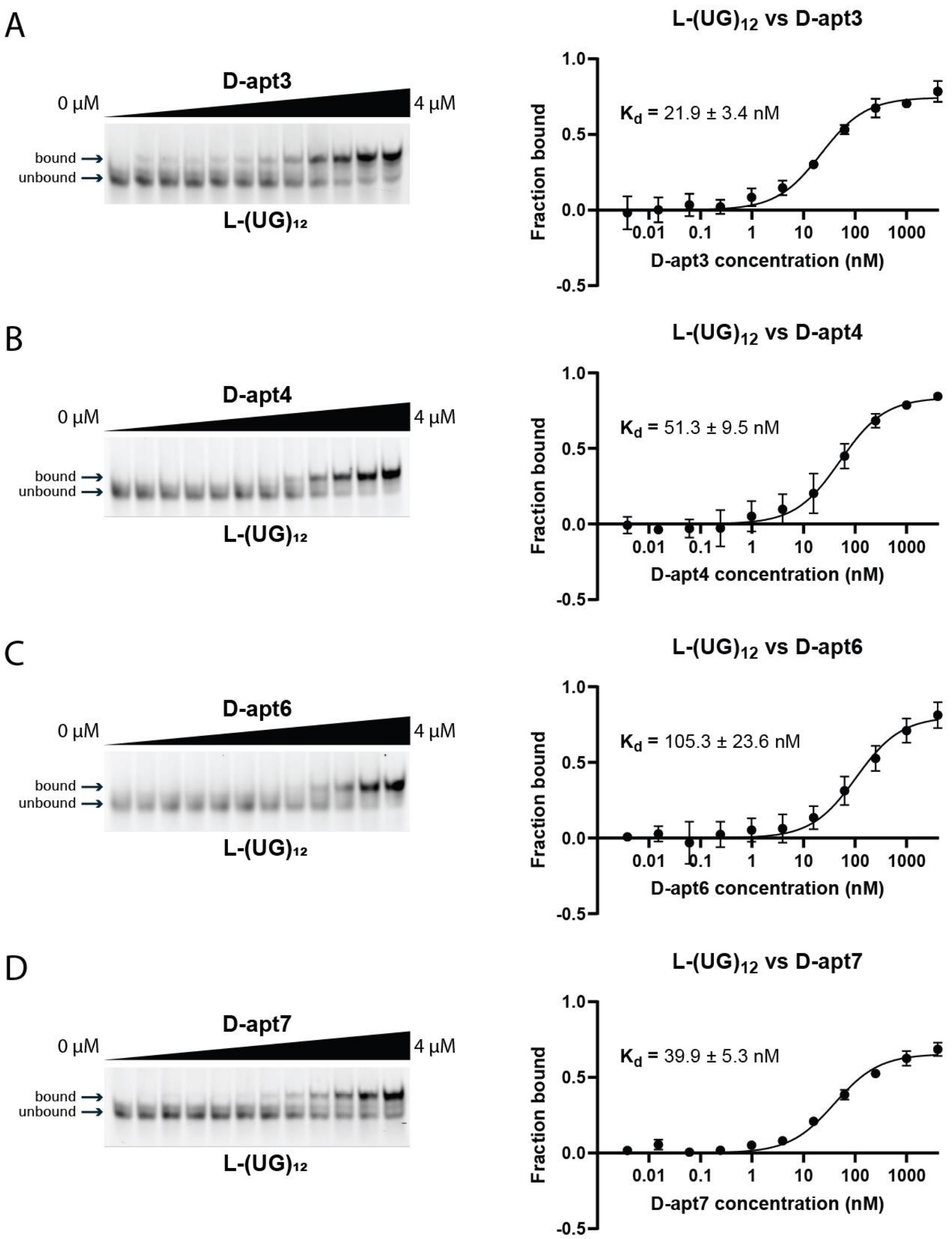

Figure S6. The binding between FAM-L-$(UG)_{12}$ and the aptamer candidates with stronger binding via EMSA. (A) Binding with D-apt3. The Kd value was determined to be 21.9 ± 3.4 nM. Data is representative of three independent experiments presented as mean ± s.d. (B) Binding with D-apt4. The Kd value was determined to be 51.3 ± 9.5 nM. (C) Binding with D-apt6. The Kd value was determined to be 105.3 ± 23.6 nM. (D) Binding with D-apt7. The Kd value was determined to be 39.9 ± 5.3 nM. D-apt3 was selected for further analysis due to its lowest Kd value. Data is representative of two independent experiments presented as mean ± s.d for (B)-(D).

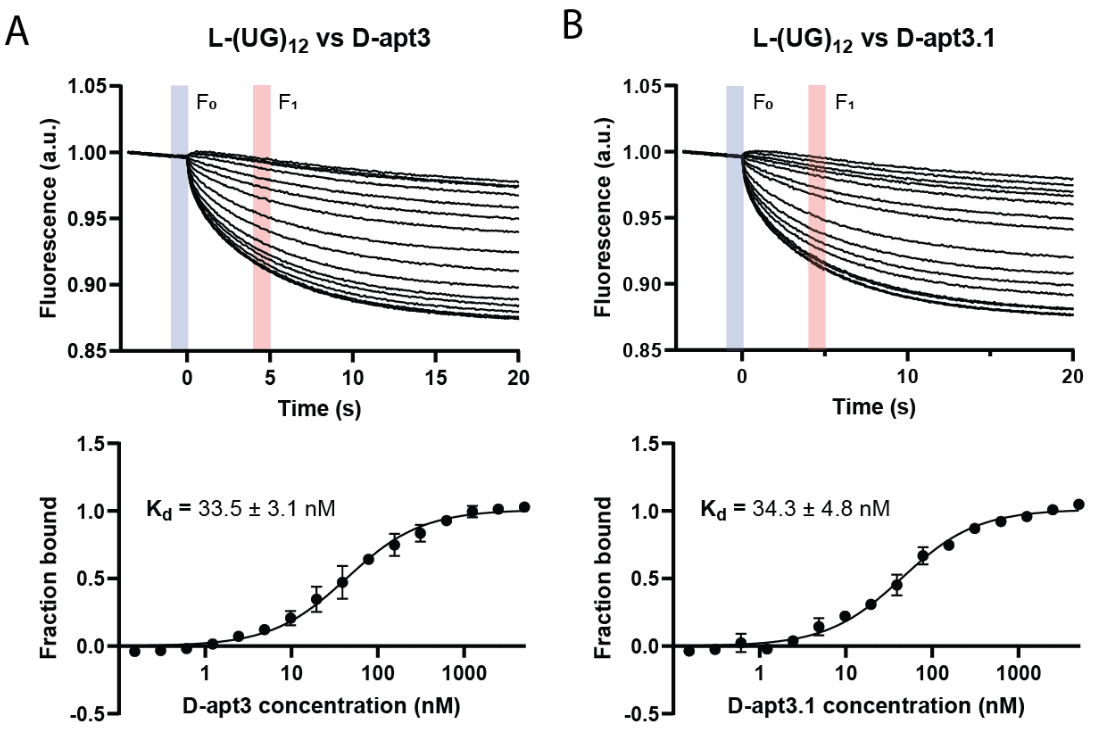

Figure S7. The binding of FAM-L-$(UG)_{12}$ against D-apt3 and D-apt3.1 via MST. The top panels show the raw fluorescent time traces while the bottom panels show the binding curves. F_0 is the initial fluorescent intensity before the temperature change is applied during the MST measurement, while F_1 is the fluorescent intensity of a defined time interval after the temperature is applied for the MST measurement. The binding curve was plotted with the normalized fluorescent intensity ($F_{norm} = F_1/F_0$) against the logarithm of aptamer concentration. (A) Binding with D-apt3. The Kd was calculated to be 33.5 ± 3.1 nM. Data is representative of three independent experiments presented as mean ± s.d. (B) Binding with D-apt3.1. The Kd was calculated to be 34.3 ± 4.8 nM. The Kd values for L-$(UG)_{12}$ against both D-apt3 and D-apt3.1 were determined to be similar, which correspond with the EMSA results for L-$(UG)_{12}$ against D-apt3 and D-apt3.1 (Fig. 1D). Data is representative of three independent experiments presented as mean ± s.d.

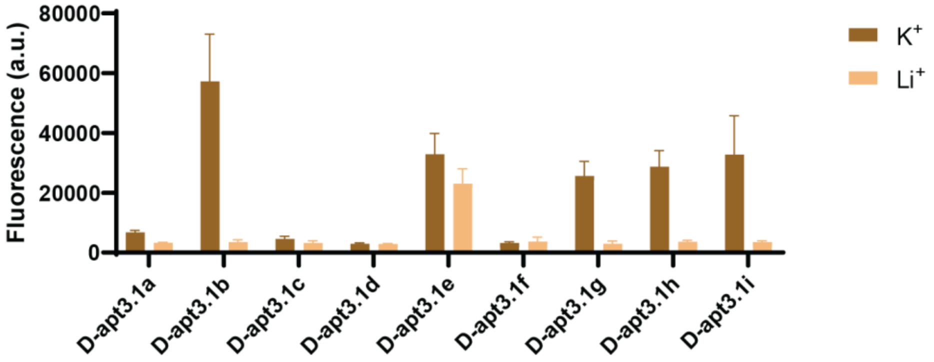

Figure S8. NMM enhanced fluorescence spectroscopy validates the presence of rG4 structure in D-apt3.1 mutants. All L-$(UG)_{12}$-binding mutants consist of an rG4 structure, which is represented by the higher fluorescent intensity in K^+ condition. Most mutants that do not bind to L-$(UG)_{12}$ possess a non-rG4 structure, with similar signals found between K^+ and Li^+ conditions, except for D-apt3.1b, which most likely adopts a different rG4 configuration from D-apt3.1.

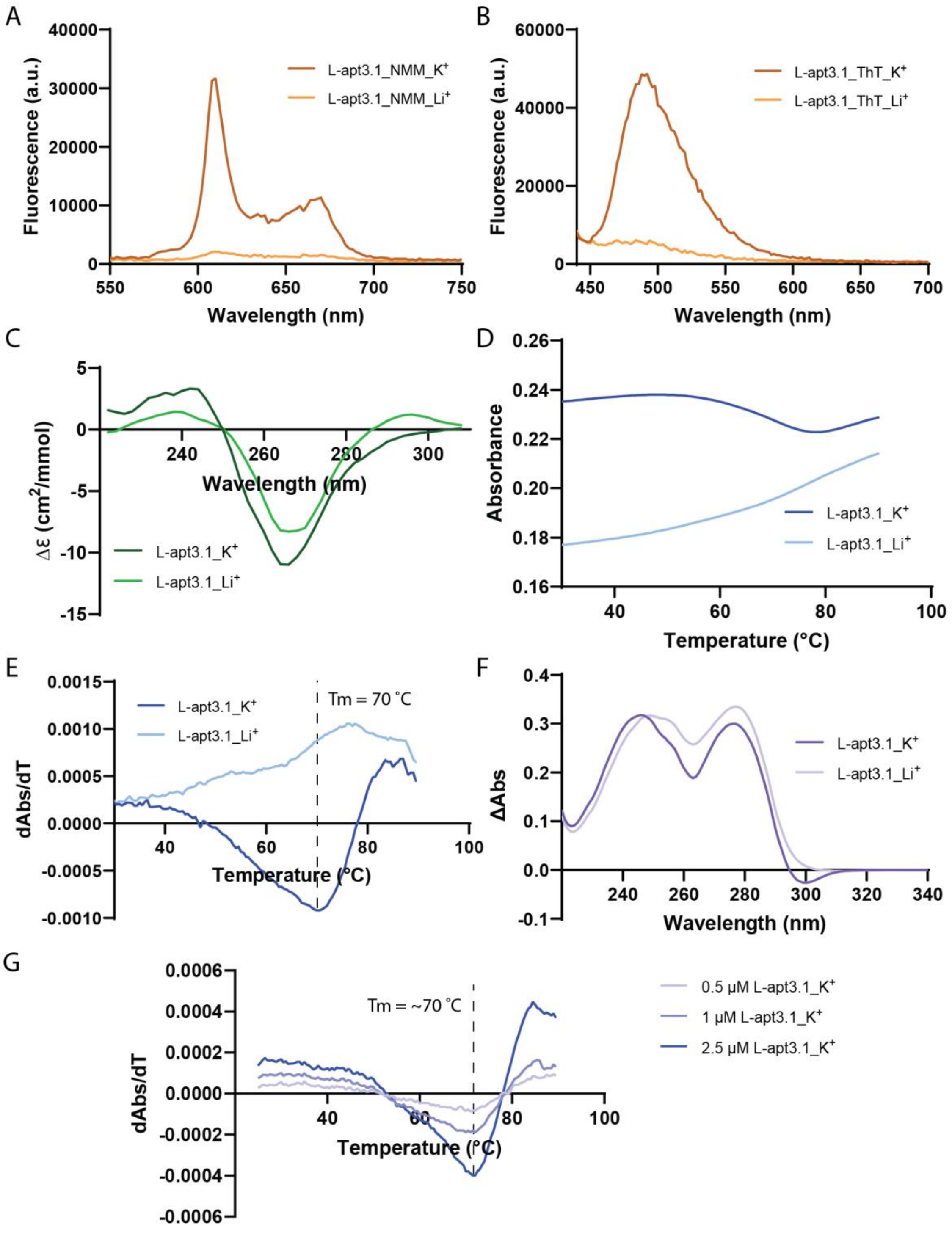

Figure S9. Structural analysis of L-apt3.1 shows that L-apt3.1 shares an identical configuration with opposite chirality as D-apt3.1 (Fig. 2). (A) NMM and (B) ThT enhanced fluorescence spectroscopies show a higher fluorescence in the presence of K^+, suggesting the formation of an rG4 structure in L-apt3.1. (C) CD spectrum demonstrates an inverted trend as compared to the spectrum of D-apt3.1 (positive peak at 242 nm, negative peak at 268 nm), indicating the parallel rG4 topology of L-apt3.1. (D) First derivative UV melting curve shows a decrease in absorbance at a wavelength of 295 nm, which is attributed by the denaturation of rG4 structure in L-apt3.1. (E) UV melting spectrum further supports that L-apt3.1 shares an identical

configuration with D-apt3.1, by which both D-apt3.1 and L-apt3.1 have a Tm of 70 °C. (F) TDS spectrum of L-apt3.1 is similar to that of D-apt3.1, with positive peaks at 246 nm and 276 nm, and a negative peak at 300 nm, indicating the high similarities between the two structures. (G) Concentration dependent UV melting spectrum reveals a similar Tm of L-apt3.1 at different concentrations, indicating the formation of an intramolecularly folded rG4 structure in L-apt3.1.

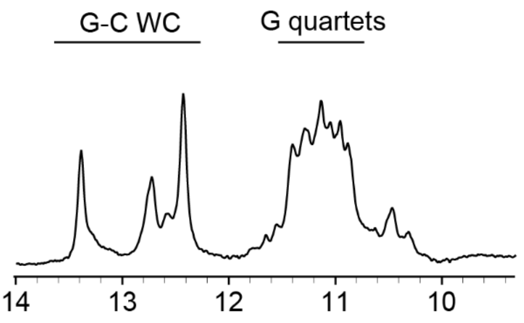

Figure S10. 1D NMR analysis of L-apt3.1. The imino resonances between 12-14 ppm are consistent with formation of the central G-C Watson-Crick pairs in the S1 helix. The resonances between 10.5-11.5 ppm are consistent with a Hoogsteen hydrogen bonded network of G quartets.

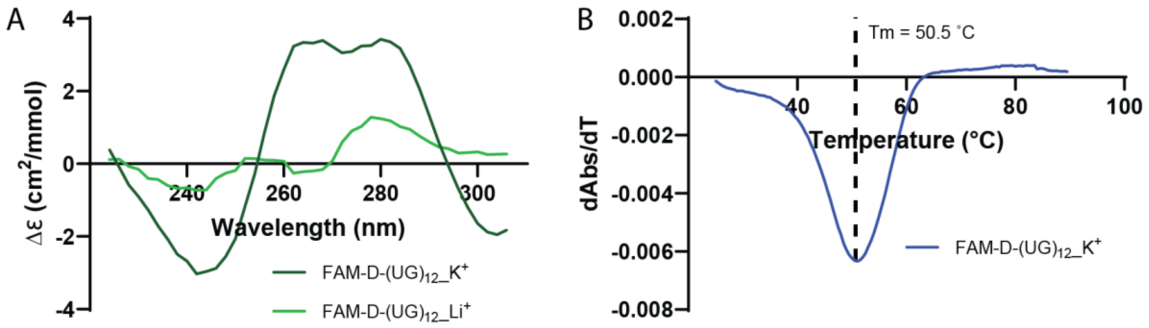

Figure S11. Spectroscopic analysis of FAM-D-$(UG)_{12}$ reveals that the FAM label does not affect the pUG fold structure folding. (A) CD spectrum of FAM-D-$(UG)_{12}$ is similar to that of unlabelled D-$(UG)_{12}$ (Fig. S1A), with signals higher under K^+ condition, and negative peak at 242 nm and positive doublets at 262 and 280 nm. (B) UV melting spectrum. Tm was determined to 50.5 °C, which is the identical to that of unlabelled D-$(UG)_{12}$ (Fig. S1B).

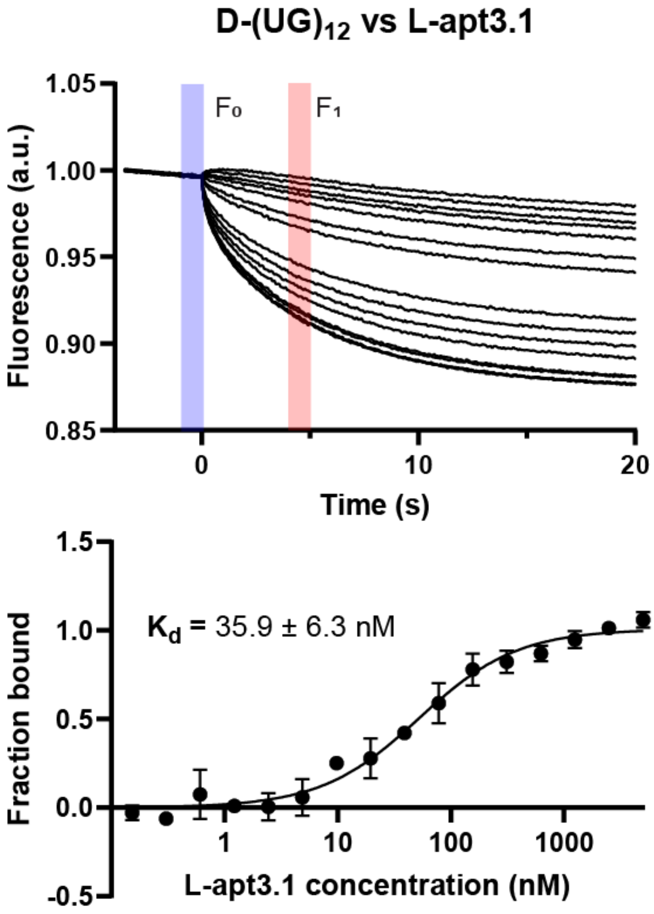

Figure S12. The binding between FAM-D-$(UG)_{12}$ and L-apt3.1 via MST. The top panels show the raw fluorescent time traces while the bottom panels show the binding curves. F_0 is the initial fluorescent intensity before the temperature change is applied during the MST measurement, while F_1 is the fluorescent intensity of a defined time interval after the temperature is applied for the MST measurement. The binding curve was plotted with the normalized fluorescent intensity ($F_{norm} = F_1/F_0$) against the logarithm of aptamer concentration. The Kd value was determined to be 35.9 ± 6.3 nM, which is similar to that of L-$(UG)_{12}$ against D-apt3.1 (Fig. S5), indicating that inversion of the chirality does not affect binding, where $(UG)_{12}$ can interact strongly with apt3.1 of the opposite chirality. Data is representative of three independent experiments presented as mean ± s.d.

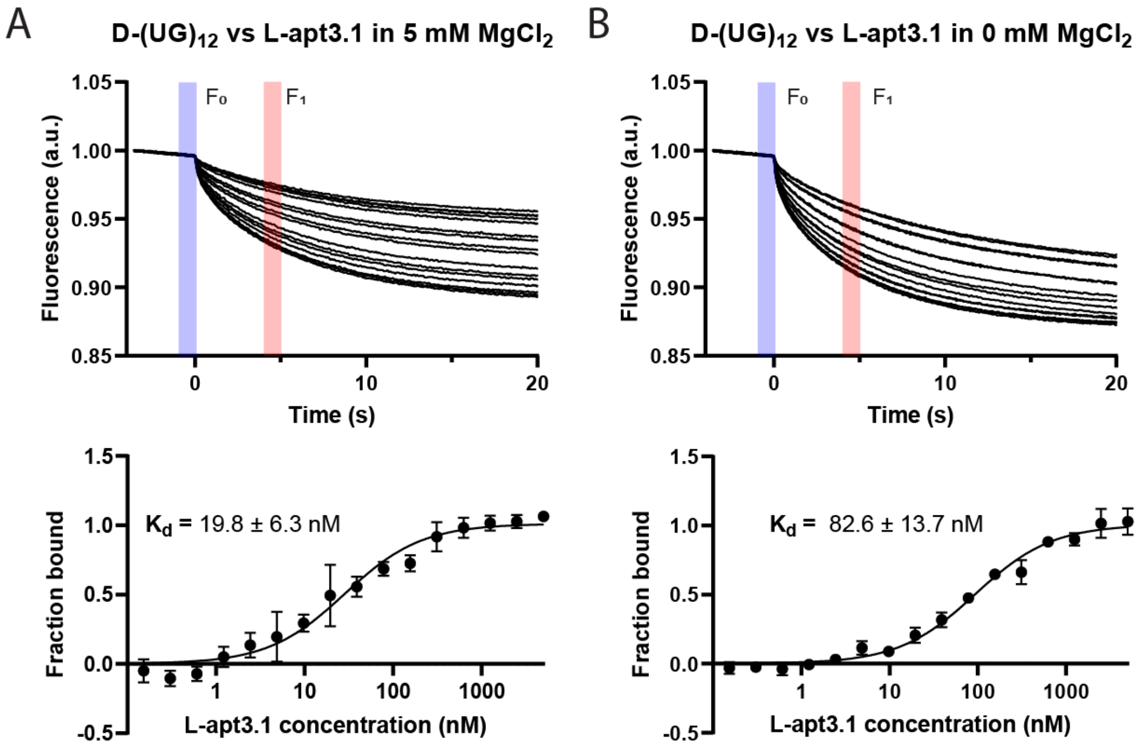

Figure S13. $MgCl_2$ dependency test for the binding between FAM-D-$(UG)_{12}$ and L-apt3.1 via MST. The top panels show the raw fluorescent time traces while the bottom panels show the binding curves. F_0 is the initial fluorescent intensity before the temperature change is applied during the MST measurement, while F_1 is the fluorescent intensity of a defined time interval after the temperature is applied for the MST measurement. The binding curve was plotted with the normalized fluorescent intensity ($F_{norm} = F_1/F_0$) against the logarithm of aptamer concentration. (A) The binding under 5 mM $MgCl_2$ condition. The Kd value was determined to be 19.8 ± 6.3 nM, which is around 2-fold lower than under 1 mM $MgCl_2$ condition (Fig. S6). (B) The binding under 0 mM $MgCl_2$ condition. The Kd value was determined to be 82.6 ± 13.7 nM, which is around 4-fold higher than under 1 mM $MgCl_2$ condition (Fig. S6).

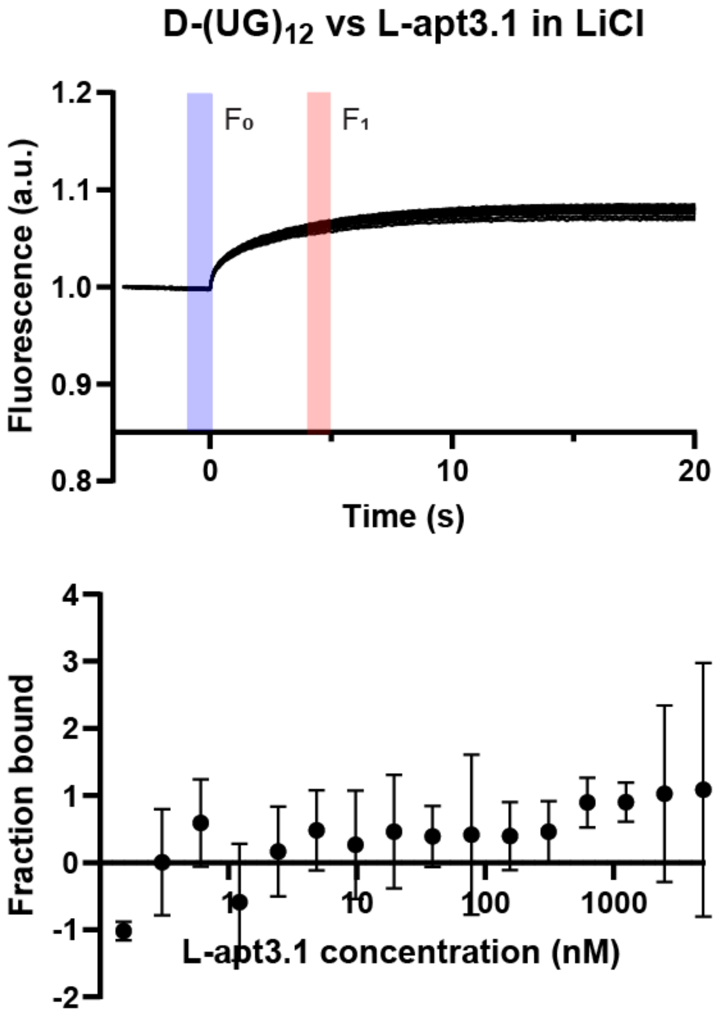

Figure S14. The binding between FAM-D-$(UG)_{12}$ and L-apt3.1 under Li^+ condition via MST. The top panels show the raw fluorescent time traces while the bottom panels show the binding curves. F_0 is the initial fluorescent intensity before the temperature change is applied during the MST measurement, while F_1 is the fluorescent intensity of a defined time interval after the temperature is applied for the MST measurement. The binding curve was plotted with the normalized fluorescent intensity ($F_{norm} = F_1/F_0$) against the logarithm of aptamer concentration. The binding curve could not be plotted due to the random dots, indicating that L-apt3.1 could not bind to D-$(UG)_{12}$ in Li^+ condition. The nonbinding is likely due to the requirement of K^+ to stabilize the rG4 motif in D-$(UG)_{12}$ and L-apt3.1.

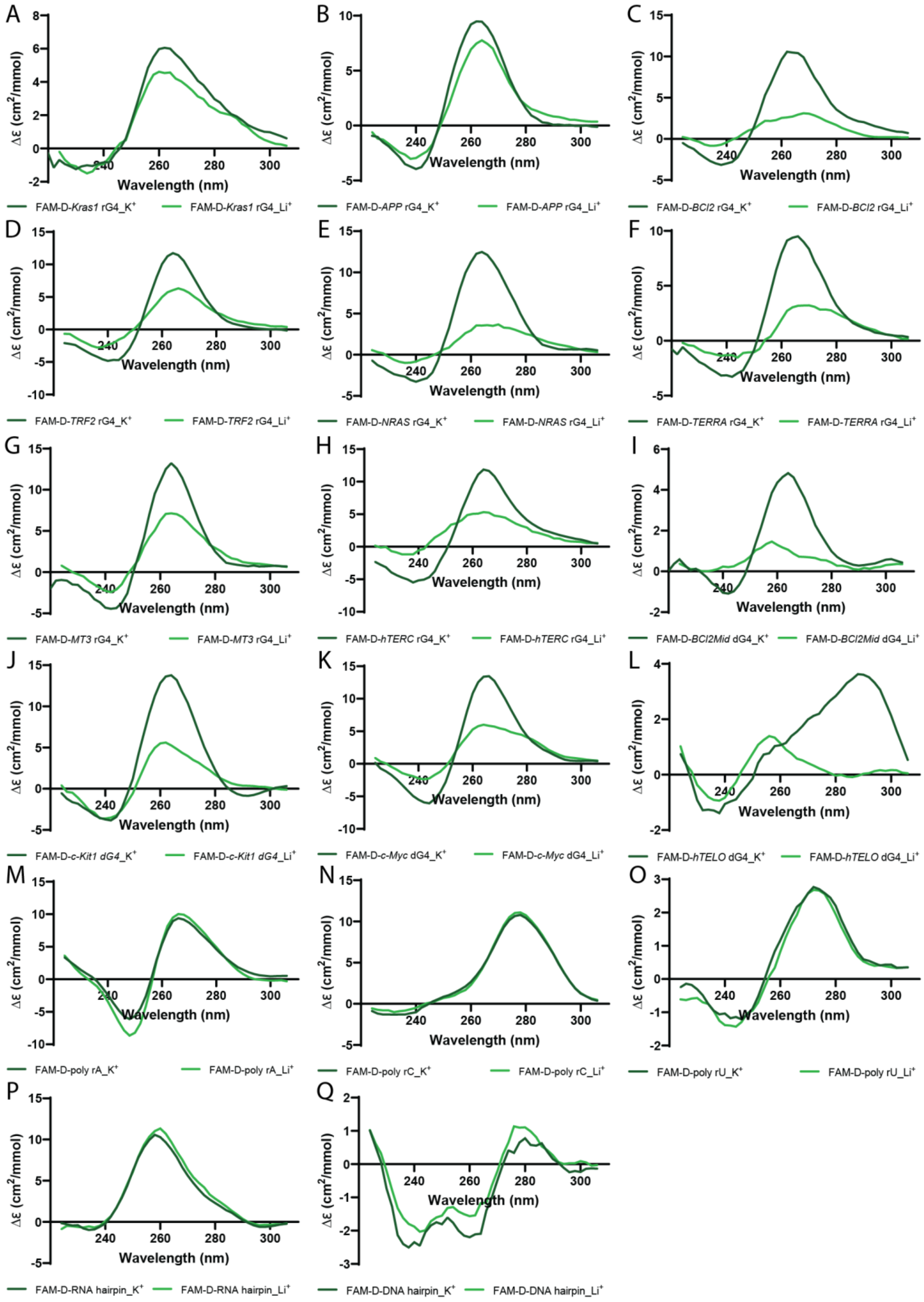

Figure S15. CD spectra to verify the presence or absence of G4 structure in the FAM-labelled off-target structural motifs. Higher signals under K^+ conditions were observed for all rG4 motifs, including (A) FAM-D-*Kras1* rG4, (B) FAM-D-*APP* rG4, (C) FAM-D-*BCl2* rG4, (D)

FAM-D-*TRF2* rG4, (E) FAM-D-*NRAS* rG4, (F) FAM-D-*TERRA* rG4, (G) FAM-D-*MT3* rG4, and (H) FAM-D-*hTERC* rG4, indicating the presence of rG4 structure. All rG4 motifs possess a parallel topology, characterized by a negative peak around 240 nm and a positive peak around 260 nm in their CD spectra. The CD spectra of all dG4 motifs, including (I) FAM-D-*BCl2mid* dG4, (J) FAM-D-*c-Kit1* dG4, (K) FAM-D-*c-Myc* dG4, and (L) FAM-D-*hTELO* dG4, also show the formation of dG4 structures, with a greater signal in the presence of K^+. The dG4 motifs either adopt a parallel topology, or a hybrid topology indicated by the negative peak around 240 nm and the positive peaks around 260 nm and 295 nm. No evidence of the formation of G4 structure was observed in all non-G4 motifs, including (M) FAM-D-poly rA, (N) FAM-D-poly rC, (O) FAM-D-poly rU, (P) FAM-D-RNA hairpin, and (Q) FAM-D-RNA hairpin, with a similar signal observed for both K^+ and Li^+ conditions.

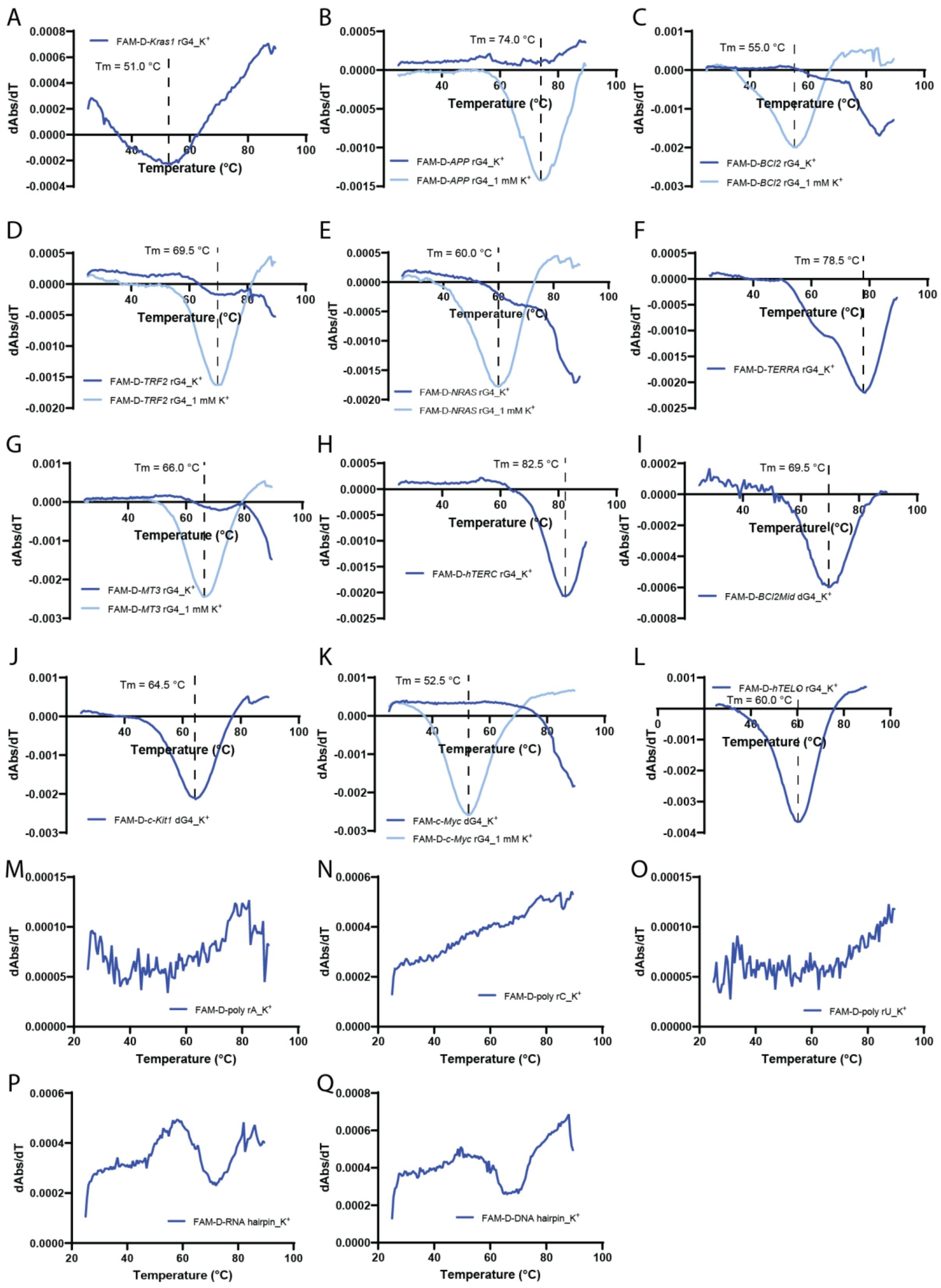

Figure S16. UV melting spectra to verify the presence or absence of G4 structure in the FAM-labelled off-target structural motifs. All spectra were assessed at a wavelength of 295 nm with 150 mM K^+ unless specified. All rG4 motifs, including (A) FAM-D-*Kras1* rG4, (B) FAM-D-

APP rG4, (C) FAM-D-*BCl2* rG4, (D) FAM-D-*TRF2* rG4, (E) FAM-D-*NRAS* rG4, (F) FAM-D-*TERRA* rG4, (G) FAM-D-*MT3* rG4, and (H) FAM-D-*hTERC* rG4, as well as all dG4 motifs, including (I) FAM-D-*BCl2mid* dG4, (J) FAM-D-*c-Kit1* dG4, (K) FAM-D-*c-Myc* dG4, and (L) FAM-D-*hTELO* dG4, demonstrate the presence of G4 structure. As observed in the hypochromic shifts, the Tms of the G4 motifs range from 51 °C to over 95 °C at 150 mM K^+ condition. (M) FAM-D-poly rA, (N) FAM-D-poly rC, (O) FAM-D-poly rU, (P) FAM-D-RNA hairpin, and (Q) FAM-D-RNA hairpin.

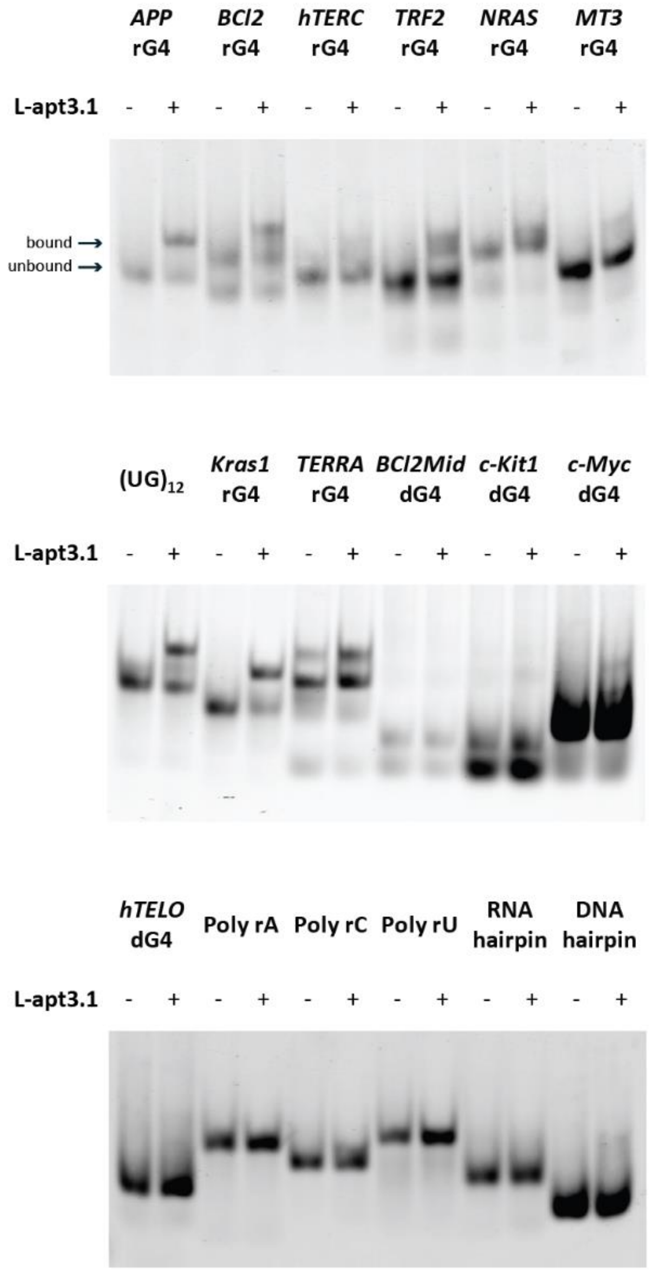

Figure S17. Unsliced gel for the selectivity test for L-apt3.1 against other FAM-labelled structural constructs. The sliced gel is shown in Figure 4B. Each lane is either with (+) or without (-) L-apt3.1 in the presence of respective structural constructs. L-apt3.1 does not bind any non-rG4 motifs, and it can bind to a few other rG4 motifs.

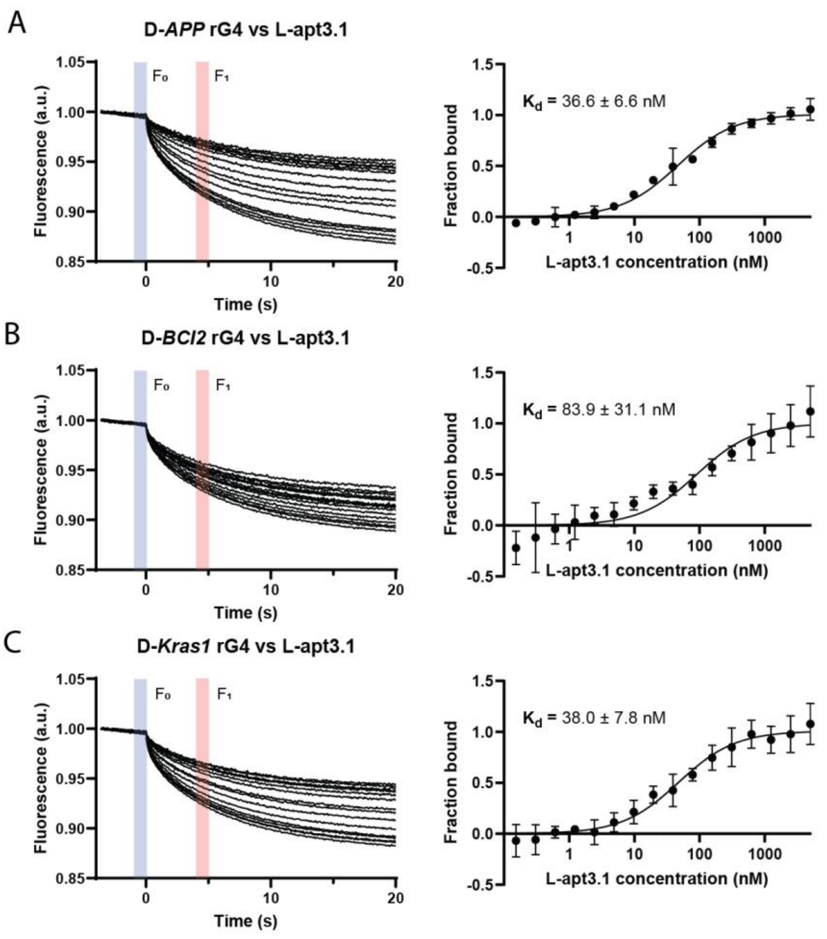

Figure S18. The binding between L-apt3.1 and 3 other FAM-labelled rG4 motifs via MST. The top panels show the raw fluorescent time traces while the bottom panels show the binding curves. F_0 is the initial fluorescent intensity before the temperature change is applied during the MST measurement, while F_1 is the fluorescent intensity of a defined time interval after the temperature is applied for the MST measurement. The binding curve was plotted with the normalized fluorescent intensity ($F_{norm} = F_1/F_0$) against the logarithm of aptamer concentration. (A) The binding with FAM-D-APP rG4. The Kd value was determined to be 36.6 ± 6.6 nM. (B) The binding with FAM-D-BCl2 rG4. The Kd value was determined to be 83.9 ± 31.1 nM. (C) The binding with FAM-D-Kras1 rG4. The Kd value was determined to be 38.0 ± 7.8 nM. The Kd values of the three rG4 motifs are similar with D-$(UG)_{12}$, which has a Kd of 35.9 ± 6.3 nM (Fig. S6).

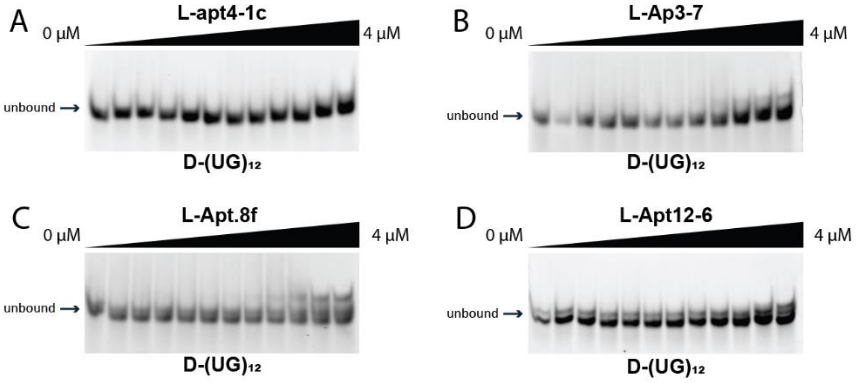

Figure S19. The binding between FAM-D-$(UG)_{12}$ and other known rG4-targeting L-RNA aptamers via EMSA. (A) Binding with L-apt4-1c, an L-RNA aptamer targeting *hTERC* rG4. (B) Binding with L-Ap3-7, an L-RNA aptamer targeting *TERRA* rG4. (C) Binding with L-Apt.8f, an L-RNA aptamer targeting *APP* rG4. (D) Binding with L-Apt-12-6, an L-RNA aptamer targeting *c-Kit1* dG4. All aptamers show weak/no binding to D-$(UG)_{12}$.

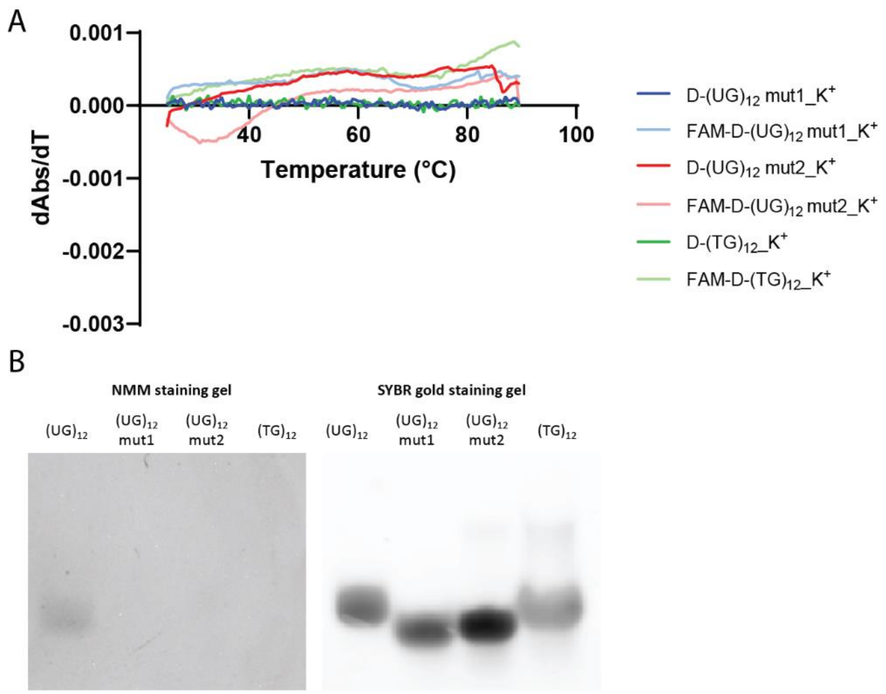

Figure S20. Spectroscopic analysis of D-$(UG)_{12}$ mutants to evaluate the presence of G4 structures. (A) UV melting spectrum. All mutants, regardless with or without FAM label, did not show any hypochromic shift at the wavelength of 295 nm, suggesting that no G4 structures are formed in the mutants. The UV melting spectrum of $(UG)_{12}$ can be obtained in Figure S1, which showed a hypochromic shift that supports the presence of G4 structure. (B) NMM staining gel and SYBR gold staining gel. NMM staining gel only shows the oligos with G4 structure presented while SYBR gold staining gel shows all presented oligos. The absence of bands in the NMM staining gel for all mutants further supports that all mutants do not consist of G4 structures.

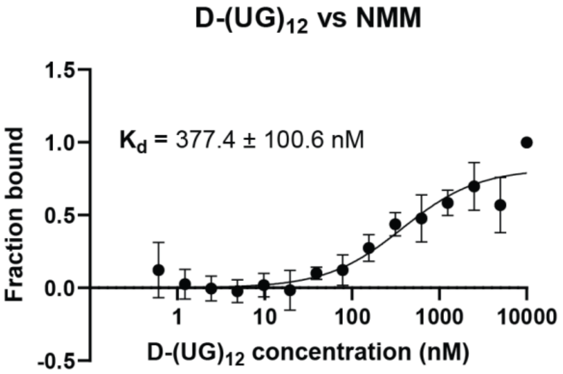

Figure S21. The binding between D-$(UG)_{12}$ and NMM via microplate reader. The Kd value was determined to be 377.4 ± 100.6 nM, which is around 15-fold higher than that with L-apt3.1 (Fig. 4A), demonstrating a preferential binding of D-$(UG)_{12}$ over L-apt3.1 than NMM.

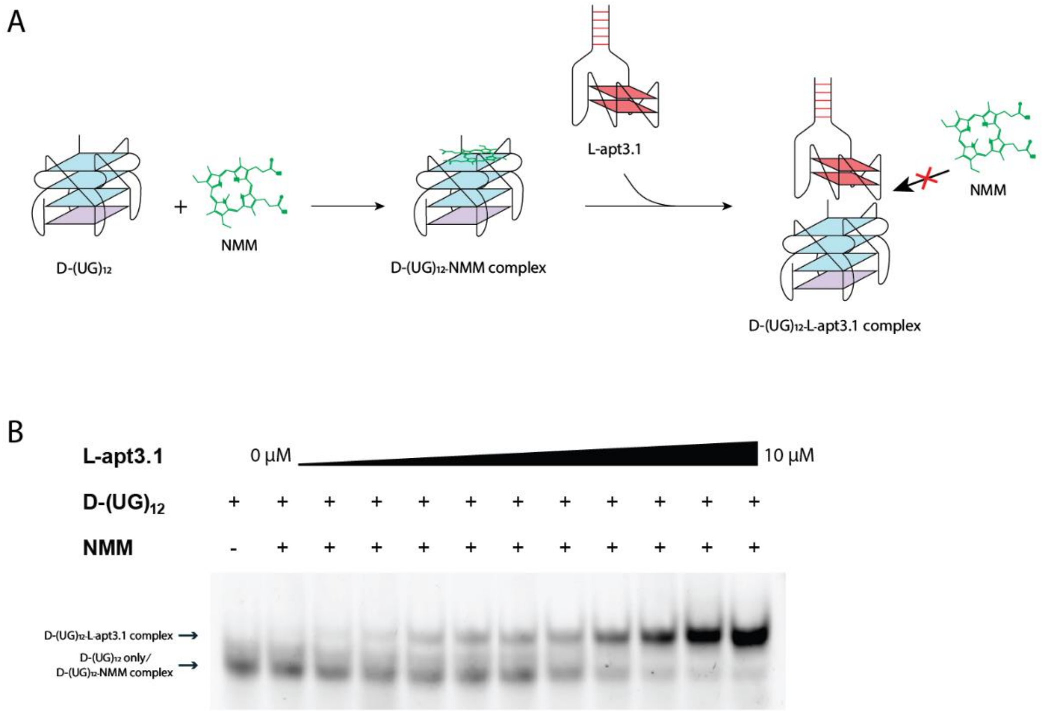

Figure S22. Displacement assay shows L-apt3.1 competing with NMM to bind with D-$(UG)_{12}$. (A) Schematic diagram of the displacement assay, where L-apt3.1 displaces NMM from D-$(UG)_{12}$. (B) The displacement assay was studied using EMSA, where L-apt3.1 displaced NMM even at low concentrations.

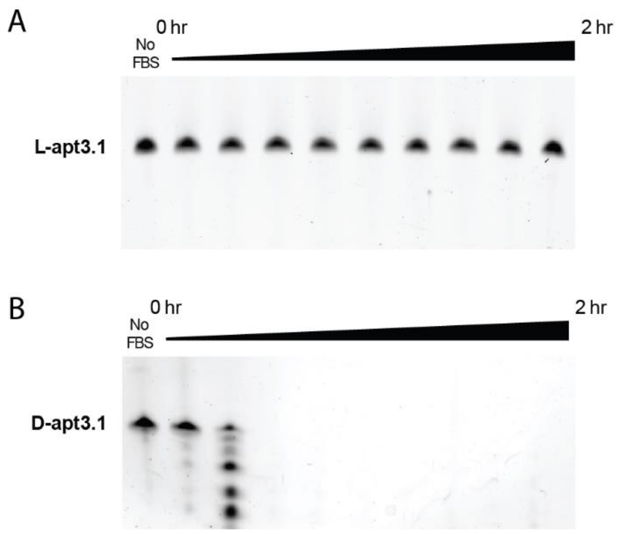

Figure S23. Stability test demonstrates the excellent stability of L-apt3.1 in biological milieu. (A) Stability test of L-apt3.1 in 5% FBS. No degradation of L-apt3.1 was observed for up to 2 hr, illustrating the great biostability of L-apt3.1. (B) Stability test of D-apt3.1 in 5% FBS. D-apt3.1 started to degrade in the first 15 min, and completely degraded in 30 min.

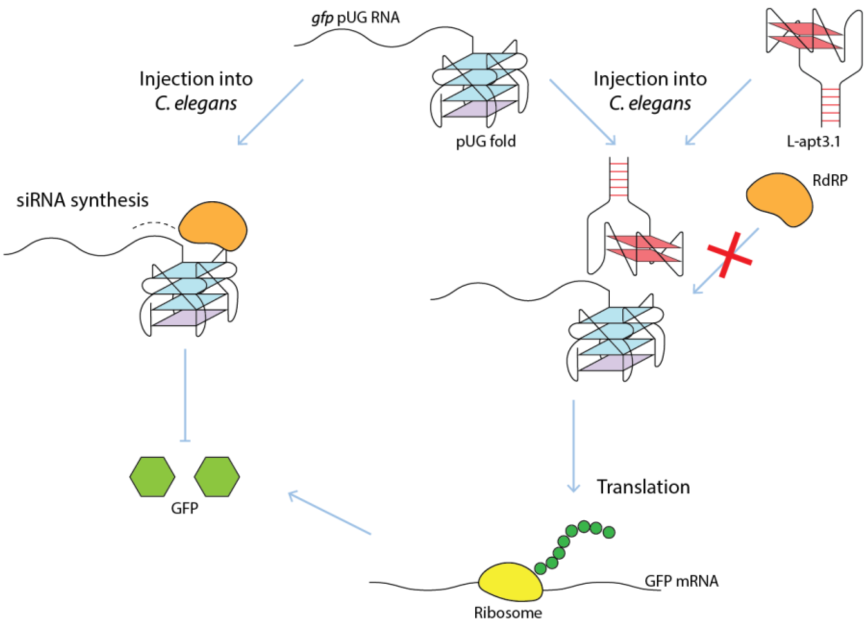

Figure S24. Schematic diagram of the mechanism of gene silencing inhibition by L-apt3.1. upon injection of the *gfp* pUG RNA consisting of the first 369 nt of the GFP coding sequences appended by $(UG)_{18}$, RdRP binds to the pUG fold and induce siRNA synthesis, hence leading to GFP gene silencing. On the other hand, the injection of the *gfp* pUG RNA and L-apt3.1 can inhibit the gene silencing from occurring, where L-apt3.1 binds to the pUG fold and prevents the recruitment of RdRP.

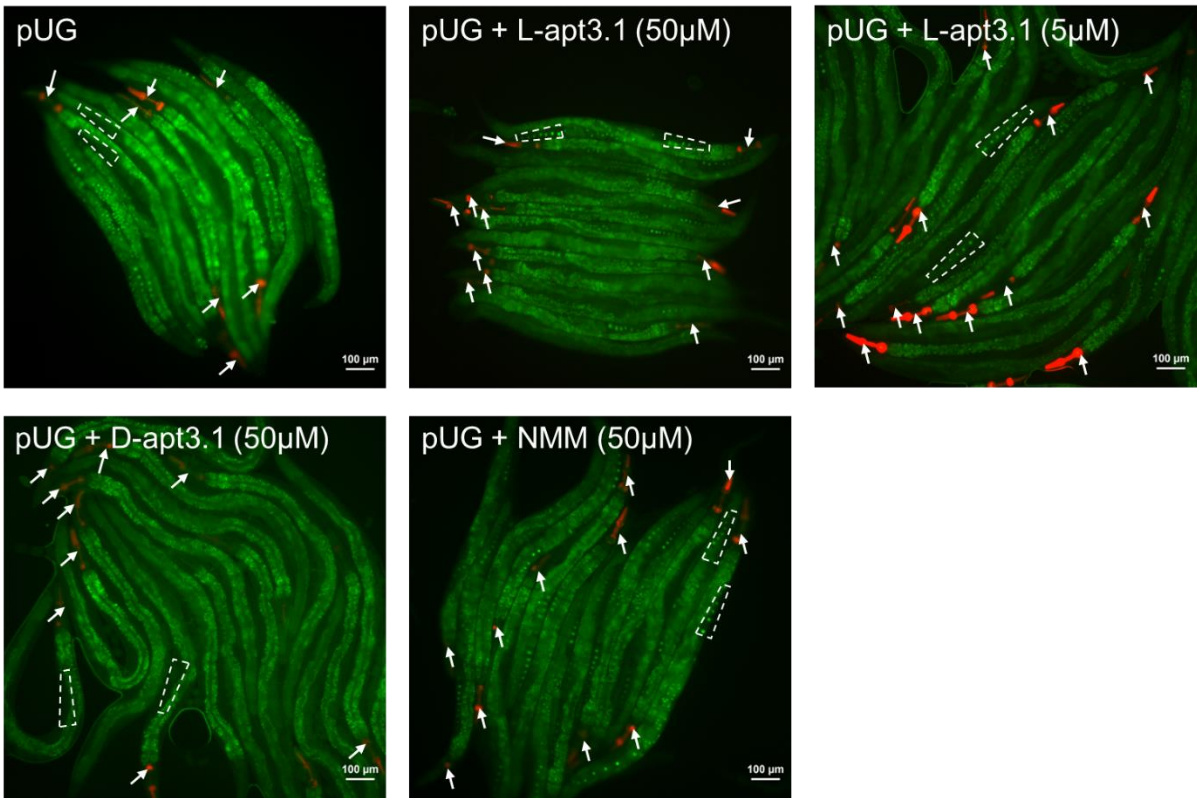

Figure S25. Representative images showing GFP expression patterns after pUG injection together with indicated molecules observed under 10× lens. White arrows mark worms expressing the injection marker plasmid (*myo-2p::mCherry*) in the pharynxes, indicating successful incorporation of injected material in these worms. Only injection marker positive worms were used for GFP expression evaluation. If not silenced, GFP is expressed in the nuclei of oocytes, showing as arrays of green dots. Dashed polygons mark some areas to check GFP expression. Note that in pUG only group and pUG + D-apt3.1 (50μM) group, GFP expression was silenced in almost all of injection marker positive worms. While in the other three groups, many injection marker positive worms still showed GFP expression.

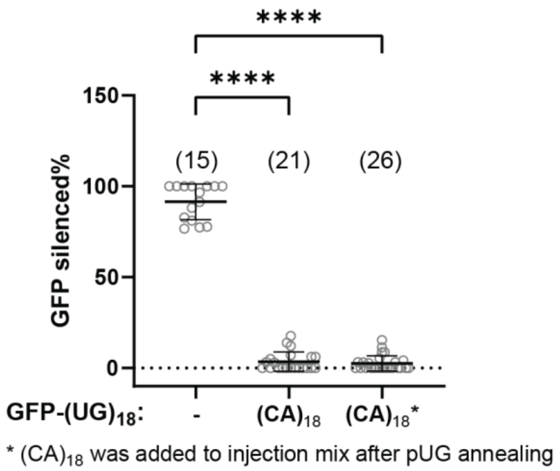

Figure S26. GFP silencing efficiency of GFP pUG RNA mixed with $(CA)_{18}$. $(CA)_{18}$ demonstrates a complete inhibition of the silencing activity whether added to injection mix before or after pUG annealing. Data are from three independent experiments presented as mean ± s.d. Numbers in parentheses are the numbers of injected P0 worms. (****P < 0.0005 for *$(CA)_{18}$; ****P < 0.0005 for $(CA)_{18}$ (one-way ANOVA)).

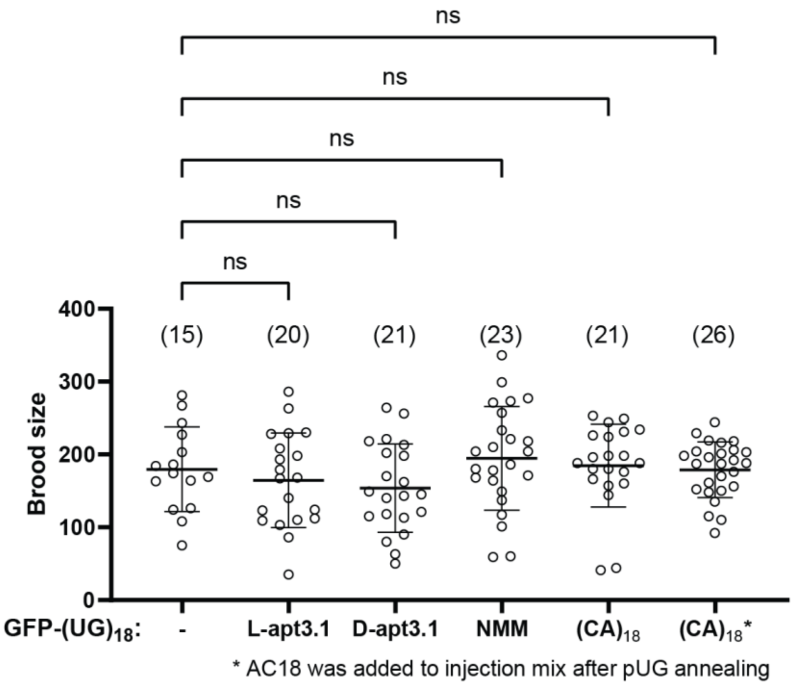

Figure S27. Brood size assay reveals that all injected molecules exhibited a negligible toxicity effect towards *C. elegans*. There were no significant differences in the brood sizes of the germlines injected with indicated molecules (L-apt3.1, D-apt3.1, NMM, $(CA)_{18}$) compared to those without injection. These results suggest that these molecules do not induce toxicity in *C. elegans*. Data are from three independent experiments presented as mean ± s.d. Numbers in parentheses are the numbers of injected P0 worms. (ns, $P > 0.05$ ($P = 0.8991$ (L-apt3.1); ns $P < 0.005$ ($P = 0.5247$ (D-apt3.1); ns $P < 0.005$ ($P = 0.8749$ (NMM)); ns $P < 0.0005$ ($P = 0.9990$ for $(CA)_{18}$; ns $P < 0.0005$ ($P > 0.9999$ for $(CA)_{18}$* (one-way ANOVA)).
